# *In Silico* Analysis of the Enzymes Involved in Haloarchaeal Denitrification

**DOI:** 10.3390/biom11071043

**Published:** 2021-07-16

**Authors:** Eric Bernabeu, Jose María Miralles-Robledillo, Micaela Giani, Elena Valdés, Rosa María Martínez-Espinosa, Carmen Pire

**Affiliations:** 1 Biochemistry and Molecular Biology Division, Agrochemistry and Biochemistry Department, Faculty of Sciences, University of Alicante, Ap. 99, E-03080 Alicante, Spain; ebs40@alu.ua.es (E.B.); jmmr19@alu.ua.es (J.M.M.-R.); micaela.giani@ua.es (M.G.); elenaperpinya@gmail.com (E.V.); rosa.martinez@ua.es (R.M.M.-E.); 2 Multidisciplinary Institute for Environmental Studies “Ramón Margalef”, University of Alicante, Ap. 99, E-03080 Alicante, Spain

**Keywords:** denitrification, haloarchaea, structural modelling, nitrate reductase, nitrite reductase, nitrous oxide reductase

## Abstract

During the last century, anthropogenic activities such as fertilization have led to an increase in pollution in many ecosystems by nitrogen compounds. Consequently, researchers aim to reduce nitrogen pollutants following different strategies. Some haloarchaea, owing to their denitrifier metabolism, have been proposed as good model organisms for the removal of not only nitrate, nitrite, and ammonium, but also (per)chlorates and bromate in brines and saline wastewater. Bacterial denitrification has been extensively described at the physiological, biochemical, and genetic levels. However, their haloarchaea counterparts remain poorly described. In previous work the model structure of nitric oxide reductase was analysed. In this study, a bioinformatic analysis of the sequences and the structural models of the nitrate, nitrite and nitrous oxide reductases has been described for the first time in the haloarchaeon model *Haloferax mediterranei*. The main residues involved in the catalytic mechanism and in the coordination of the metal centres have been explored to shed light on their structural characterization and classification. These results set the basis for understanding the molecular mechanism for haloarchaeal denitrification, necessary for the use and optimization of these microorganisms in bioremediation of saline environments among other potential applications including bioremediation of industrial waters.

## 1. Introduction

**Figure 1:**
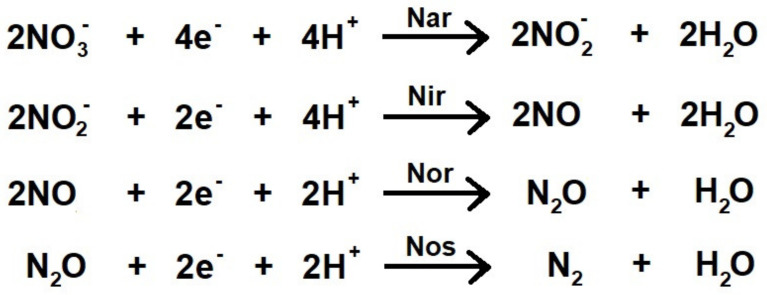
Summary of the four key reactions involved in denitrification (adapted from [4]). Nar: Respiratory nitrate reductase (EC 1.7.5.1); Nir: Respiratory nitrite reductase (EC 1.7.2.1); Nor: Respiratory nitric oxide reductase (EC 1.7.2.5); Nos: Respiratory nitrous oxide reductase (EC 1.7.2.4).

The nitrogen cycle (N-cycle) is one of the most important biogeochemical cycles which is mainly driven by prokaryotes. This cycle contains different pathways with several redox reactions that comprise assimilatory and respiratory processes for energy conservation [1]. Some of the reactions involved make denitrification the most energetically profitable out of all types of anaerobic dissimilatory pathways carried out by bacteria and archaea [2]. It consists of a microbial respiratory process in which nitrate is used as an alternative electron acceptor when soluble oxygen is not available for aerobic respiration. The whole pathway involves the sequential reduction of nitrate (NO_3_^−^) to dinitrogen (N_2_) via nitrite (NO_2_^−^), nitric oxide (NO), and nitrous oxide (N_2_O) [3] (Figure 1).

Briefly, two nitrate ions are sequentially reduced to N_2_ in a series of four oxidation-reduction reactions catalysed by four metalloenzymes known as respiratory nitrate reductase (Nar), respiratory nitrite reductase (Nir), nitric oxide reductase (Nor) and nitrous oxide reductase (Nos) [4]. In this process, a total of 10 electrons are transferred to nitrogen oxides to complete their reduction to dinitrogen [4]. The presence of a total or partial set of denitrification enzymes determines whether a microorganism is classified as complete or incomplete denitrifier, respectively [5,6,7,8].

Denitrification has been extensively studied in the Bacteria domain at biochemical, physiological, and structural levels [3,9,10,11,12], but the information in *Archaea* domain is still limited. The Archaea domain has attracted the attention of researchers in the last two decades due to the unique characteristics of these microorganisms that allow them to survive in extreme conditions of pH, temperature, or salinity. Haloarchaea are halophiles capable of living under different sources of stress, such as high salt concentrations, high solar radiation, and elevated temperatures [13,14,15]. The molecular mechanisms that allow adaptation to these extreme environments are of great biotechnological interest and may give rise to new lines of research. They have potential application in bioremediation processes and in the production of compounds of interest for the pharmaceutical, medical and biotechnological industries, as carotenoids and bioplastics [16]. Several well-known haloarchaeal species belonging to *Haloferax*, *Haloarcula* and *Natrinema* genera have been described as denitrifiers [8,17,18,19]. Haloarchaeal denitrifiers might be useful in bioremediation since they can remove nitrate and nitrite from soil and wastewater using these molecules as final electron acceptors [8].

Among all denitrifying haloarchaea, *Haloferax mediterranei* has become the main representative model given that it produces all required metalloenzymes (NarGH, CuNirK, qNor and NosZ) to catalyse the complete reduction of NO_3_^−^ to N_2_. Genomic organization of the genes involved in denitrification has been described [8] and the *nar* operon has been sequenced and characterized at transcriptional level [20], showing induction during oxygen-limiting and nitrate-sufficient conditions. The potential application of *Haloferax mediterranei* as a bioremediating agent in contaminated saline waters has been studied [21]. *Haloferax mediterranei* gas emission under denitrifying conditions has been measured, confirming that it is a complete denitrifier able to transform nitrate to dinitrogen even more efficiently than other *Haloferax* species [7,22]. *Haloferax mediterranei* NarGH and NirK have been studied at the biochemical level reporting expression in anaerobic conditions [20,21]. Molecular biology and microbial ecology in connection with denitrification have also been studied [23,24]. However, the denitrification pathway in archaeal domain remains poorly understood. There is enough evidence to propose that active site of nitrate reductase of *Haloferax mediterranei* is on the outside of the cytoplasmic membrane and denitrification pathway could take place between plasma membrane and the S-layer [8,21], but by the time of this work, no three-dimensional structure for any of the core denitrification enzymes is yet available. Molecular modelling has only been conducted on nitric oxide in *Haloferax mediterranei* [25]. This enzyme belongs to the long-chain respiratory NOR (lcNORs or qNORs) class, which contain a single subunit known as NorZ whose C-terminal chain acts as catalytic domain and the N-terminal chain as electron transfer domain which transports electrons from quinol. *norZ* gene is widely distributed in halophilic archaea being the only nor gene found in these microorganisms. The structural model of the enzyme from *Haloferax mediterranei* revealed that it is very similar to its bacteria counterparts, being the structure of the quinol binding site and the water channel highly conserved [25].

This article presents for the first time the complete set of in silico structural models of the remaining three main enzymes of denitrification pathway in *Haloferax mediterranei*. These results offer new opportunities to better understand the catalytic mechanisms of the enzymes from haloarchaea and may even support the design of possible mutants that improve their catalytic efficiency for biotechnological applications.

## 2. Materials and Methods

### 2.1. Selection of Species for the Analysis of Sequences Alignments

Two approaches were used to select relevant species to conduct the alignment of the sequences: i) Sequences coding for the enzymes in at least one organism from which the enzymes have been structurally characterized were included in all the alignments analysed; ii) Sequences coding for the enzymes of denitrification in species described as complete denitrifiers were included (two haloarchaea species from each family (this classification is according to Taxonomy NCBI Database). After species selection, the alignments of the sequences were performed using Clustal Omega software (https://www.ebi.ac.uk/Tools/msa/clustalo/, accessed on 18 June 2020.). This bioinformatic tool is based on the HHalgorithm described by Söding [26,27], and for these alignments default parameters were used. 

### 2.2. Sequence Analysis and Alignments

The amino acid sequences of NarG (Protein NCBI ID: WP_004056332.1), NarH (Protein NCBI ID: WP_004056335.1), NirK (Protein NCBI ID: WP_004059594.1) and NosZ (Protein NCBI ID: WP_004056356.1) from *Haloferax mediterranei* were analysed using SignalP-5.0 Server (http://www.cbs.dtu.dk/services/SignalP/, accessed on 26 November 2020) and ScanProsite tool (https://prosite.expasy.org/scanprosite/, accessed on 25 November 2020) and TatLipo Server (http://signalfind.org/tatlipo.html, accessed on 26 November 2020) in order to determine TAT, TAT/Lipo signal sequences and 4Fe-4S domains [28,29]. Theoretical pI was obtained using ProtParam from Expasy (https://web.expasy.org/protparam/, accessed on 22 June 2020).

### 2.3. Model Building

Homology modelling was carried out using the SWISS-Model software (https://swissmodel.expasy.org/, accessed on 8 November 2020) using default modelling parameters [30,31]. The quality of the model was measured using GMQE and QMEAN parameters. GMQE (Global Model Quality Estimation) provides quality estimation which combines properties from the target-template alignment and the template structure. This estimation reflects the expected accuracy of a model built with that alignment and template, normalized by the coverage of the target sequence. Values of QMGE around zero indicates low quality whereas higher numbers (around one) indicate higher reliability. QMEAN provides an estimation of the degree of nativeness of the structural features of the model compared to what would be expected from the experimental structures [32,33]. Values of QMEAN around zero indicate good quality models meanwhile scores of −4.0 or below indicate low quality. Models was carried out using mature protein sequences (excluding TAT signal peptide). Cofactors have been included in the model structure at the same site than in the templates. Orientations and distances are not optimized with respect to the residues in the model. The calculated distances of the interaction between the atoms in the model and in the cofactors are compared with those in the templates in Table S1.

### 2.4. Analysis of Electrostatic Surface Potential and Illustration

Models structure analysis and illustrations were carried out using PyMol Molecular Graphics System, Version 2.3.2 Schrödinger, LLC. Cofactors were incorporated into the finished homology model at the same site of templates. The residue numbering of all alignments includes the N-terminal signal peptide region. Mature enzymes were used (excluding N-terminal signal peptide region) to carry out the model building. However, the model numbering contains N-terminal signal peptide region to be in concordance with alignments. The electrostatic surface analysis was performed using Adaptative Poisson-Boltmann Solver plug-in in PyMol software (Version 2.3.2 Schrödinger, LLC.)

## 3. Results and Discussion

### 3.1. Respiratory Nitrate Reductase

Respiratory nitrate reductases (Nar) belong to the dimethyl sulfoxide (DMSO) reductase family (EC 1.8.5.3), and are characterized by the presence of a molybdenum (Mo) atom located in the active site. Bacterial Nar enzymes are heterotrimeric complexes that comprise three structural subunits, NarG, NarH and NarI. NarG (112–140 kDa) is the catalytic subunit (α-subunit) that contains a molybdenum *bis* molybdopterin guanine dinucleotide (Mo-*bis*-MGD) cofactor in its catalytic site and iron–sulphur cluster [4Fe-4S] (FS0) for electron transfer [11,34]. This polypeptide chain can be divided into five domains: four involved in Mo-bis-MGD binding, and a fifth domain involved in the diffusion of the substrate and product from the active site [10,11]. NarH (52-64 kDa) is the electron transfer subunit (β-subunit) which contains three [4Fe-4S] (FS1, FS2 and FS3) and one [3Fe-4S] cluster (FS4). Each cluster is coordinated by cysteine residues in several species [10,11,35]. The NarH core structure shows two domains (A and B), each one holding one high-potential and one low-potential cluster (FS1:FS2 and FS3:FS4, respectively). NarI (19-25 kDa) is the integral membrane subunit (ϒ-subunit) composed by five transmembrane α-helices. This subunit anchors NarGH to the membrane and contain two haem *b* groups that participate in the oxidation of ubiquinol [11].

In *Haloferax mediterranei* genome the respiratory nitrate reductase gen cluster is in the plasmid pHM300. It contains genes coding for the catalytic and the electron transfer subunit, NarG and NarH, respectively, but there is no a gene that encode a product similar to bacterial NarI [8,20].

#### 3.1.1. N-terminal Analysis Exposes Conflict of Annotation in Several Haloarchaeal Species

**Figure 2:**
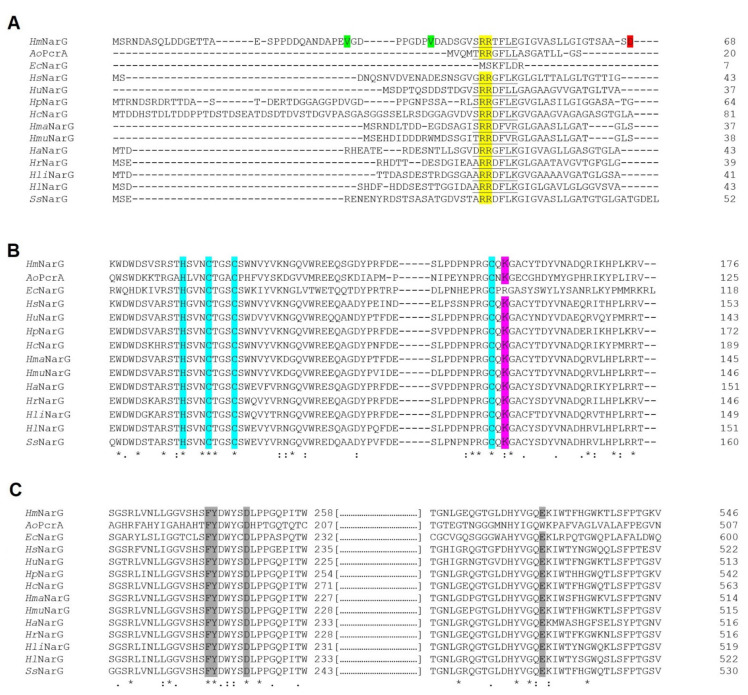
Clustal omega alignment of *Azospira oryzae* (*Ao*PcrA; Protein NCBI ID: WP_014235273.1), *Escherichia coli* (*Ec*NarG; Protein NCBI ID: WP_000032939.1), *Halococcus salifodinae* (*Hs*NarG; Protein NCBI ID: EMA48671.1), *Halorhabdus utahensis* (*Hu*NarG; Protein NCBI ID: WP_012795090.1), *Haloferax mediterranei* (*Hm*NarG; Protein NCBI ID: WP_004056332.1), *Halogeometricum pallidum* (*Hp*NarG; Protein NCBI ID: WP_008385261.1), *Halosimplex carlsbadense* (*Hc*NarG; WP_006885135.1), *Haloarcula marismortui* (*Hma*NarG; Protein NCBI ID: WP_011223493.1), *Halomicrobium mukohataei* (*Hmu*NarG; Protein NCBI ID: WP_015763420.1), *Halogranum amylolyticum* (*Ha*NarG; Protein NCBI ID: WP_089826390.1), *Halorientalis regularis* (*Hr*NarG; Protein NCBI ID: WP_092694237.1), *Halorubrum lipolyticum* (*Hli*NarG; Protein NCBI ID: WP_008003395.1), *Halobellus limi* (*Hl*NarG; Protein NCBI ID: SEF60617.1), and *Salinigranum salium* (*Ss*NarG; Protein NCBI ID: WP_152042447.1). (**A**) N-terminal signal peptide region. The TAT consensus sequence [ST]RRxFLK is underlined, twin arginine ‘RR’ is represented in yellow, hypothetical start codons and the first amino acid of mature of *Haloferax mediterranei* NarG are shown in green and red, respectively. (**B**) Metal-coordinating regions of FS0 cluster. His and Cys residues involved in FS0 cluster coordination are highlighted in cyan whereas Lys involved in a possible electron transfer pathway between FS0 and MoCo cofactor are rendered in magenta. (**C**) NarG active site. The conserved residues involved in catalytic mechanism are highlighted in grey.

In the N-terminal signal peptide region of *Haloferax mediterranei* NarG, a distinctive twin-arginine translocase (TAT) exportation motif ([ST]RRxFLK) has been identified (Figure 2A), which is absent in the same region in NarH. This difference indicates that folded NarGH dimer might be transported across the plasma membrane by the TAT system. The TAT signal peptide is a short N-terminal motif characterized by the presence of a positive N-terminal region, a hydrophobic region and a consensus short motif AxA which is the specific cleavage site for a signal peptidase [36,37]. The TAT system transports folded proteins across the plasma membrane in *Bacteria* and *Archaea* and it plays a critical role in protein transport in haloarchaea [21,38,39,40]. 

All the NarG enzymes from haloarchaea present the TAT signal sequence in the N-terminal region (Figure 2A). In *Haloferax mediterranei* NarG, the predicted protease cleavage site is between Ser_67_ and Asp_68_ (AAS-DD), therefore, the mature NarG would begin with an aspartate residue. The size of the *Haloferax mediterranei* NarG signal peptide is 67 residues, which is larger than those identified in the rest of analysed species except for *Halogeometricum pallidum* and *Halosimplex carlsbadense*. This feature could be due to ambiguous annotation. It is important to note that this conflict of annotation has been previously described in other haloarchaeal studies [25,41]. It is possible to assume that *narG* start codon was GUG instead of AUG, being the first amino acid Val_30_ or Val_38_, (Figure 2A). GUG start codon is less frequent than AUG in prokaryotes, nevertheless, its presence is relevant in genomes with high GC content such as the *Haloferax mediterranei* genome [42]. 

#### 3.1.2. The Structural Model Shows Spatial Conservation of All Residues Involved in Cofactors Binding

To obtain an insight into the structure of the respiratory nitrate reductase from *Haloferax mediterranei,* a homology modelling study was conducted. The best template was the structure of the perchlorate reductase (PcrAB) from *Azospira oryzae* (PDB: 4ydd.1.AB). For NarG modelling, the sequence without the TAT signal peptide was used as query, showing a sequence identity with PcrA of 45%, and a coverage of 95%. The quality of the model was supported by a QMEAN of -2.21 and a QMQE of 0.77. NarH reported an identity of 51%, with a coverage of 93% with PcrB and the QMEAN and QMQE were of −0.46 and of 0.75, respectively. NarG structure was modelled from Pro_16_ to Asp_916_ (numbering according to mature protein which corresponds to residues 83 and 983 in WP_004056332.1). NarH structure was modelled from Asp_17_ to Asp_350_ (which maintains the same amino acid numbering in WP_004056335.1). 

**Figure 3:**
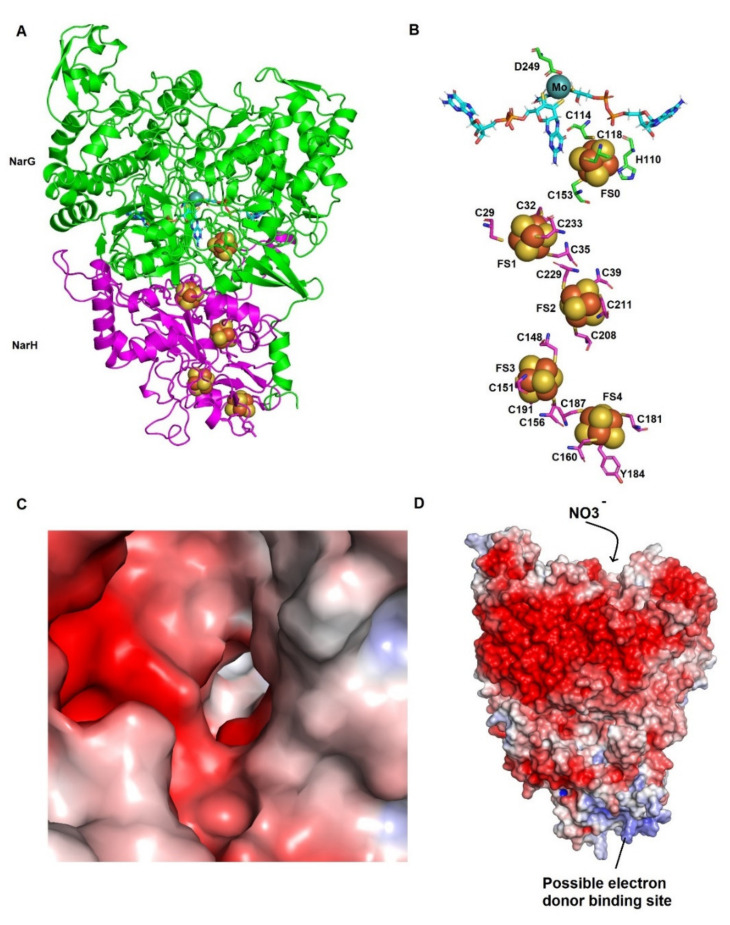
Homology model and electrostatic surface potential of *Haloferax mediterranei* NarGH. (**A**) Overall structural model of the *Haloferax mediterranei* NarGH dimer. NarG subunit is coloured in green cartoon and NarH subunit is coloured in magenta cartoon. Iron-sulphur clusters are shown as orange and yellow spheres, respectively. Mo-*bis*-MGD cofactor is represented as cyan sticks whereas molybdenum atom is coloured in turquoise. (**B**) Relative position of cofactors in *Haloferax mediterranei* NarGH. Amino acids involved in cofactor binding are shown as sticks. (**C**) Electrostatic surface potential of tunnel in the *Haloferax mediterranei* NarGH. (**D**) Electrostatic surface potential of *Haloferax mediterranei* NarGH dimer. The electrostatic potential is represented between +5 and −5 *kT/e*.

The structural model of NarGH from *Haloferax mediterranei* is shown in Figure 3. The largest NarG catalytic subunit conserves the characteristic structure of the Moco active site and the [4Fe–4S] centre (FS0). The NarH is the electron transfer subunit and conserve the residues that ligate the three [4Fe–4S] centres (FS1, FS2, FS3) and the [3Fe–4S] centre (FS4).

In DMSO reductase family members, the Mo atom of the MoCo is ligated, in a trigonal prismatic manner, by four thiolates of the bis-MGD cofactor, an inorganic ion like oxygen, sulphur, or selenium atoms and an amino acid ligand, such as serine, cysteine, selenocysteine or aspartate residues. The nature of this ligand is the basis for the classification of the three types of DMSO reductases. Respiratory nitrate reductases have an aspartate residue as the Mo ligand, like the other members of type II group of the DMSO reductase family [10,43]. The architecture of the MoCo cluster in *Haloferax mediterraeni* could be similar to the one observed in *Azospira oryzae* PcrA being Asp_249_ the Mo ligand. This Asp residue, involved in catalysis, is homologous to Asp_198_ in PcrA and Asp_222_ in *Escherichia coli* NarG. In *Azospira oryzae* PcrA, the sixth ligand is an oxygen atom from an oxo, hydroxyl, or associated water molecule as in NarG from *E.coli* [10,35].

Regarding the iron-sulphur clusters in *Haloferax mediterranei* NarGH model, all [Fe-S] clusters are coordinated by four cysteine residues with exception of the [4Fe–4S] center (FS0) of NarG subunit. The specific pattern for FS0 coordination CxxCxxC(x)nC has been reported in formate dehydrogenases (Fdh), periplasm nitrate reductases (Nap) and citoplasmic assimilatory nitrate reductases (Nas). However, in NarG the FS0 cluster is ligated by three Cys side residues and by one His sidechain. This coordination pattern HxxxCxxxC(x)nC seems to be conserved in all type II DMSO reductases (Figure S1) [10,35] and other iron-sulphur clusters of ‘Rieske’ proteins [44].

**Figure 4:**
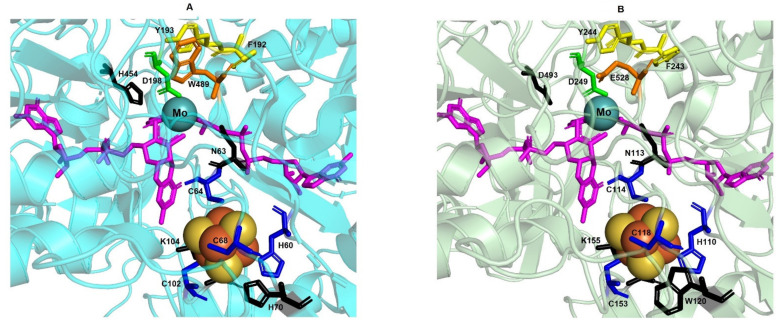
Crystal structure of *Azospira oryzae* PcrA (PDB ID: 4YDD; (**A**) and homology model of *Haloferax mediterranei* NarG (**B**). The PcrA and NarG catalytic subunits appear in cartoon form and are coloured in light blue and olive, respectively. Cysteines and histidine residues (dark blue sticks) involved in the coordination of the FS0 cluster (Iron and sulphur as orange and yellow spheres, respectively) are conserved in both structures. The Mo atom is a turquoise sphere and bis-MGD cofactor is represented with magenta sticks. The aspartate ligand that coordinates the Mo atom in the active site (Asp_198_ and Asp_249_ in PcrA and NarG, respectively) is shown as a green stick. The gate putative residues (Phe_192_ and Tyr_193_ in PcrA and Phe_243_ and Tyr_244_ in NarG) are shown as yellow sticks. Orange sticks indicate non-homologous amino acid substitution of tunnel gates (Trp_489_ for PcrA and Glu_528_ for NarG). Black sticks represent amino acids with possible role in substrate binding (N_63_ and N_113_ in PcrA and NarG, respectively), reduction potential regulation of FS0 (H_70_ and W_120_ in PcrA and NarG, respectively), electron transport bridge between FS0 and MOCO cofactor (K_104_ and K_155_ in PcrA and NarG, respectively), and proton donor during catalysis (H_454_ and D_493_ in PcrA and NarG, respectively).

This coordination pattern is also observed in *Haloferax mediterranei* NarG (residues His_110_, Cys_114_, Cys_118_, Cys_153_) (Figure 2B and Figure S1) and in other haloarchaea. Alignments presented here suggest that all analysed haloarchaea NarG sequences contain this coordination pattern, except for *Halobiforma haloterrestris* and *Halopiger aswanensis* (Figure 2B). The cluster coordination by the His chain has been suggested to increase the reduction potential [44]. The location of FS0, between Mo-bis-MGD and FS1 cluster in NarH, has been hypothesized to play a direct role in the electron transfer mechanism. In fact, a mutagenesis study in which this His is mutated to Cys resulted in the loss of NarGHI activity in *Escherichia coli* [45]. The close location between the FS0 cluster and the cofactor in both *Haloferax mediterranei* NarG and PcrA suggests the presence of a similar pathway of electron transfer to the catalytic site despite 46.04% sequence similarity. In *E.coli* NarGH the loop in which the FS0 ligands are found also include an Asn that could be implicated in substrate binding, and a Trp that is bonded to the S4 in FS0 cluster [10]. Both residues are also conserved in a similar orientation in NarG model from *Haloferax mediterranei* (Asn_113_ and Trp_120_) but in *Azospira oryzae* PcrA, the Trp residue is substituted by His. It has been proposed that the hydrogen bond between Trp and FS0 may increase the reduction potential of the FS0 cluster [10]. As well, a conserved Arg, which is near the FS0 cluster, could act increasing the reduction potential in *Escherichia coli* NarG [10]. Interestingly, the corresponding amino acid is a Lys in *Azospira oryzae* PcrA and haloarchaea NarG (Lys_155_ in *Haloferax mediterranei* NarG) (Figure 4 and Figure S1) as well as in periplasmatic Naps (data not shown).

With regard to the catalytic mechanism, it has been described that the oxidation state of the Mo ion changes between Mo (VI) and Mo (V) during the oxo-transfer reaction, and an oxygen ligand (OH/H_2_O) is released upon cofactor reduction to Mo (IV). EPR Mo (V) signals revealed that NarG displays low-pH and high-pH forms. In the *Escherichia coli* NarG structure, Asp_222_ is hydrogen bonded to the N of a conserved His_546_, which would be deprotonated in the transition to the high-pH form [10]. One of the two protons involved in the nitrate reduction might be this one associated with the acid-base transition between low-pH and high-pH forms. The His residues, highly conserved in bacterial nitrate reductases is an Asp in halophilic nitrate reductases (Asp_493_ in *Haloferax mediterranei* NarG). The absence of this exchangeable proton with the medium indicates that the proton-transfer mechanism associated with the nitrate reduction could be different, as occur with periplasmic nitrate reductase [10], in which the residue equivalent to His is Gln.

In *Escherichia coli* NapA no solvent-exchangeable protons are detected by EPR in the Mo (V) state, but the catalytic mechanism is similar than in NarG. It seems that Mo (VI) is ligated to a water molecule, which is lost on reduction to Mo (V) allowing the nitrate binding. The Mo (V)-nitrate is then reduced by an electron arising from the FS0 centre to Mo (IV) and subsequent the nitrate is reduced to nitrite. The bounded oxygen of nitrate is then transferred to the molybdenum as a terminal oxo group, which protonate to give the stable Mo (VI)–OH_2_ [46,47]. In *Desulfovibrio desulfuricans* NapA a slightly different mechanism occurs in which nitrate binds to the Mo (IV) form [48]. The catalytic mechanisms in these enzymes are yet not clear and alternative pathways for the reduction of nitrate have been postulated depending on pH and substrate concentration in which nitrate can alternatively binds to Mo (IV) and Mo (V) forms [49]. The catalytic mechanism in *Haloferax mediterranei* should be assess by protein-film voltammetry and EPR experiments.

Although the electron transfer pathway between NarH and NarG in halophilic archaea could be similar to that described in bacteria, the orientation in the membrane is inverted and a membrane anchoring subunit similar to the bacterial NarI is not conserved [21]. As the enzyme from *Haloferax mediterranei* has been isolated as a dimer NarGH, it is not clear the identity of the electron donor to NarH [20]. In Figure 3C,D, the surface electrostatic potential of active site tunnel and NarGH are shown. As expected, the surface shows a high negative charge density, which is a general characteristic of halophilic enzymes with a high percentage of acidic residues in surface, likewise the contact surface with the electron donor subunit is easily located as it has neutral and positive charge density in surface. The product of gene *Orf7* of the nitrate reductase cluster could be the electron donor to NarH [20]. This protein is similar to soluble *b*-type cytochromes encoded in the operons of other type II molybdoenzyme, for example SerA in the *Thauera selenatis* selenate reductase and EbdC in the *Aromatoleum aromaticum* ethylbenzene dehydrogenase subunit [21]. A trimer structure of NarGH with the protein encoded by *Orf7* has been modelling using as template the EdbC form *Aromatoleum aromaticum* (36.65% of sequence identity, GMQE and QMEAN of 0.63 and −3.17 respectively), showing that this trimer could be established (data not shown). Nevertheless, in a recent proteomic study of denitrification in *Haloferax mediterranei*, this protein was not identified, and it was postulated that NarH could interact with NarC, which is homologous to the cytochrome *b* subunit of cytochrome *bc1* complex. Further experiments should be done to confirm the identity of the electron donor to NarH.

Miralles-Robledillo and coworkers reported that haloarchaeal NarG is closer to (per)chlorate reductases (PcrA) group than to bacterial NarG [43]. Despite this fact, NarG from haloarchaea and PcrA from Bacteria are classified as two different monophyletic groups. On the one hand, PcrAB catalyses the reduction of oxo chlorates, such as perchlorate and chlorate to form hypochlorite under anaerobic conditions [50]. On the other hand, nitrate reductases (NarGH) carry out the reduction of nitrate to nitrite. However, it has been reported that *Azospira oryzae* PcrAB can recognize nitrate as substrate. Conversely, *Haloferax mediterranei* and *Haloarcula marismortui* nitrate reductases can reduce perchlorate and chlorate [18,51]. The biochemical characterization of NarGH reported that this enzyme can use oxo chlorates as substrates with an efficiency comparable to its natural substrate [21,51]. In addition, bromate and iodate have also been identified as substrates of these enzymes. When the structural model of *Haloferax mediterranei* NarG and the crystal structure of *Azospira oryzae* PcrA were compared, some differences in the substrate accessing gate tunnel and in the active site were detected (Figure 4A,B).

Figure 2C shows the alignment of the gate and the active site from the haloarchaeal NarG subunit with *Azospira oryzae* PcrA and *Escherichia coli* NarG. In this alignment, we can observe the conserved catalytic aspartate (Asp_249_) and three residues, Phe_243_, Tyr_244_, and Glu_528,_ which are part of the substrate gate tunnel. These gating residues overlay accurately with the well-studied structure of *Azospira oryzae* PcrA. Likewise, Phe_243_ and Tyr_244_ in NarG correspond to Phe_192_ and Tyr_193_ in PcrA (Figure 2C). However, a non-homologous Glu residue (Glu_528_) has been detected in NarG instead of the Trp_489_ observed in PcrA. This change was also reported in *Escherichia coli* NarG [35] and it is conserved in NarGs from bacteria (Figure S1). In PcrA catalytic subunit, the substrate access tunnel has positive charges creating a high affinity for oxyanions. Conversely, in *Haloferax mediterranei* NarG monomer the gate tunnel has predominantly negative charges (Figure 3C,D).

Mutational studies have been performed in the gate residues and in the catalytic Asp in *Azospira oryzae* PcrA to determine the impact of residues replacement on cell growth and catalytic efficiency [35]. F192A, Y193A and W489A mutations prevented growth using perchlorate as electron acceptor. Interestingly, PcrA W489E mutant retained its ability to grow with chlorate and still displayed activity to reduce perchlorate although its K_m_ was notably increased (Table 1). Nevertheless, what is more surprising and counterintuitive is that this Trp to Glu mutation greatly decreased the PcrA nitrate affinity. The authors suggested that this feature could be due to both, the flexibility of Glu residue in the modified active site because of the lack of hydrogen bonds with Ser_620;_ and the stacking interaction with Tyr_193_ [35]. However, the enzyme from *Haloferax mediterranei,* despite presenting the Glu residue at the gate tunnel, has higher affinity for nitrate compared with *Azospira oryzae* PcrAB and with *Escherichia coli* NarGHI and therefore, the Glu residue would not hinder the nitrate selection.

The role of this Glu residue in the substrate gate tunnel of nitrate reductases is not clear. It has been proposed in PcrAB the reduction of Mo (VI) to Mo (IV) shifts catalytic Asp to a bidentate coordination of the Mo (IV) leading to a conformational shift of Phe residue in the gate tunnel opening it. It is possible to assume that Glu residue acts in a similar way, preventing the premature entry of the substrate to the catalytic site and changing its conformation during catalytic mechanism. Besides, the negative charge could generate repulsion for larger anionic molecules and along with a narrow tunnel act as selective substrate filter, avoiding the binding of bulky substrates such as dimethyl sulfoxide and trimethylamine N-oxide.

### 3.2. Respiratory Nitrite Reductase

Nitrite reductases catalyse the second denitrification reaction, and they can be divided into two types of enzymes: copper-containing nitrite reductases (NirK) or cytochrome-*cd_1_*-dependent nitrite reductases (NirS) [53]. NirKs are homotrimeric enzymes (~35 kDa) which contain two copper-binding sites (type 1 and type 2 copper centre) per monomer whereas cytochrome-*cd_1_*-dependent nitrite reductases (NirS) are homodimeric and contain haems *c* and *d_1_* as cofactors [54,55]. NirK can be classified as Class 1 and Class 2. Both types are homotrimer with 2 cupredoxin domain per monomer (two-domains NirK). Class I and Class II differ in their optical absorption spectrum, with class I being blue and showing maximum absorbance at 590 nm and class II being green and with maximum absorbance at 450–460 nm [56]. New classes of NirKs have recently been identified. Class III NirKs are hexameric enzymes that contain two different type 1 copper per subunit, one of class I and one of class II [57]. Additional three-domain NirK have been identified. They contain an extra domain where *c*-type haem or cupredoxin is tethered to the N or C-terminal site [58,59,60,61]. Recently, a novel four-domain NirK has been discovered. It has been identified in species from Rhizobiales order, such as *Bradyrhizobium* sp. ORS 375 [62]. They contain the core domain of “classical” two-domains NirK (cupredoxin domain) to which both a cytochrome *c* and cupredoxin domain are tethered at the N terminus [62]. Recent studies reported a division of NirK sequences into two different phylogenetic clades. Clade I comprises mainly *Alphaproteobacteria* whereas clade II harbours more diverse taxonomic groups, including *Archaea* [56]. Each clade presents a different sequence pattern around the catalytic His: TRPHL and SSFHV/I/P.

**Figure 5:**
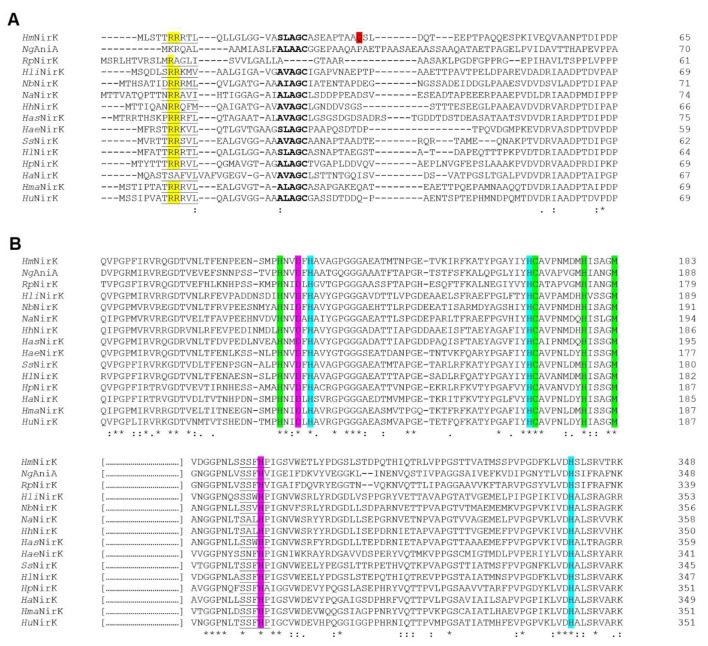
Clustal omega alignment of *Neisseria gonorrhoeae* (*Ng*AniA; Protein NCBI ID: WP_003705926.1), *Ralstonia pickettii* (*Rp*NirK; Protein NCBI ID: WP_039373687.1), *Halorubrum lipolyticum* (*Hli*NirK; Protein NCBI ID: WP_049911241.1), *Natonolimnonius barhuensis* (*Nb*NirK; Protein NCBI ID: WP_054863730.1), *Natronococcus amylolyticus* (*Na*NirK; Protein NCBI ID: WP_005555322.1), *Halobiforma haloterrestris* (*Hh*NirK; Protein NCBI ID: WP_089787040.1), *Halopiger aswanensis* (*Has*NirK; Protein NCBI ID: WP_120243376.1), *Haloplanus aerogenes* (*Hae*NirK; Protein NCBI ID: WP_121921915.1), *Salinigranum salinum* (*Ss*NirK; Protein NCBI ID: WP_152041973.1), *Halobellus limi* (*Hl*NirK; Protein NCBI ID: WP_103992402.1), *Haloferax mediterranei* (*Hm*NirK; Protein NCBI ID: WP_004059594.1), *Halogeometricum pallidum* (*Hp*NirK; Protein NCBI ID: WP_049916781.1), *Halogranum amylolyticum* (*Ha*NirK; Protein NCBI ID: WP_170864849.1), *Haloarcula marismortui* (*Hma*NirK; Protein NCBI ID: WP_011224471.1), *Halorhabdus utahensis* (*Hu*NirK; Protein NCBI ID: WP_012795101.1). (**A**) N-terminal signal peptide region. The TAT consensus sequence [ST]RRxFLK is underlined, twin arginine ‘RR’ is represented in yellow, lipoprotein consensus sequence is in bold and the first amino acid of the mature protein in *Haloferax mediterranei* is highlighted in red. (**B**) T1Cu and T2Cu coordination centres and catalytic amino acids. The catalytic amino acids are highlighted in magenta, whereas amino acids involved in T1Cu and T2Cu coordination centres are highlighted in green and cyan, respectively. The consensus sequence SSFHV/I/P is underlined.

The analysis of haloarchaeal nitrite reductases revealed that all of them are copper-containing nitrite reductases and they can be classified as two-domains NirKs containing only cupredoxin domains (Figure 5B).

#### 3.2.1. N-Terminal Inspection Reveals a TAT Signal Peptide

All haloarchaeal NirK sequences analysed show the TAT motif except from *Halogranum amylolyticum*. In addition, all of them contain the lipobox, [LVI][ASTVI][GAS]C characteristic of lipoproteins (Figure 5).

In bacteria, lipoprotein precursors are processed by signal peptidase II (SPase II), which specifically recognizes the conserved lipobox motif. A glyceride-fatty acid lipid is attached by a prolipoprotein diacylglyceryl transferase (Lgt) to the conserved cysteine of lipobox, and SPase II cleaves the precursor immediately upstream of this lipid-modified cysteine. In Gram-negative and some Gram-positive bacteria, the conserved lipobox cysteine residue is also acylated by apolipoprotein N-acyltransferase. Several bioinformatic analysis, supported by experimental data, indicate that most haloarchaeal lipoproteins are TAT substrates, in a greater proportion than in other archaea. Homologs of Lgt, SPase II and acyltransferase have not been detected in archaeal genomes, indicating that the molecular mechanisms underlying archaeal lipoprotein biosynthesis may are distinct from their bacterial counterparts [63,64].

The presence of TAT/lipobox combination indicates that halophilic NirKs could be lipoproteins exported by the TAT system through a mechanism not yet described. NirK from *Haloferax mediterranei* was overexpressed in *Haloferax volcanii* and polypeptides of different size were detected in cytoplasm and extracellular medium reporting trimeric structure [65]. The shorter isoform was predominant in cytoplasm fractions and was postulated to be the product of an alternative transcriptional processing. The longer one was the predominant in the extracellular fraction and its amino terminal sequence is not consistent with the bacterial lipoprotein processing (unfortunately the membrane fraction was not analysed) [65]. Therefore, molecular mechanisms beyond this issue remain unclear: it could be due to different processing of the polypeptide or maybe due to overexpression. In fact, both mechanisms could also work simultaneously.

The TAT motif is absent in NirK from the two bacterial species, *Ralstonia pickettii* and *Neisseria gonorrhoeae* (Figure 5A). NirK from *Ralstonia pickettii* contains a Sec-dependent signal peptide (residues 1 to 31) whereas NirK from *Neisseria gonorrhoeae*, which is an outer membrane lipoprotein, has the conserved lipobox (Figure 5A) and [66].

#### 3.2.2. The Overall Structure Is Consistent with an Homotrimer with Conserved Residues Involved in the Catalytic Mechanism

**Figure 6:**
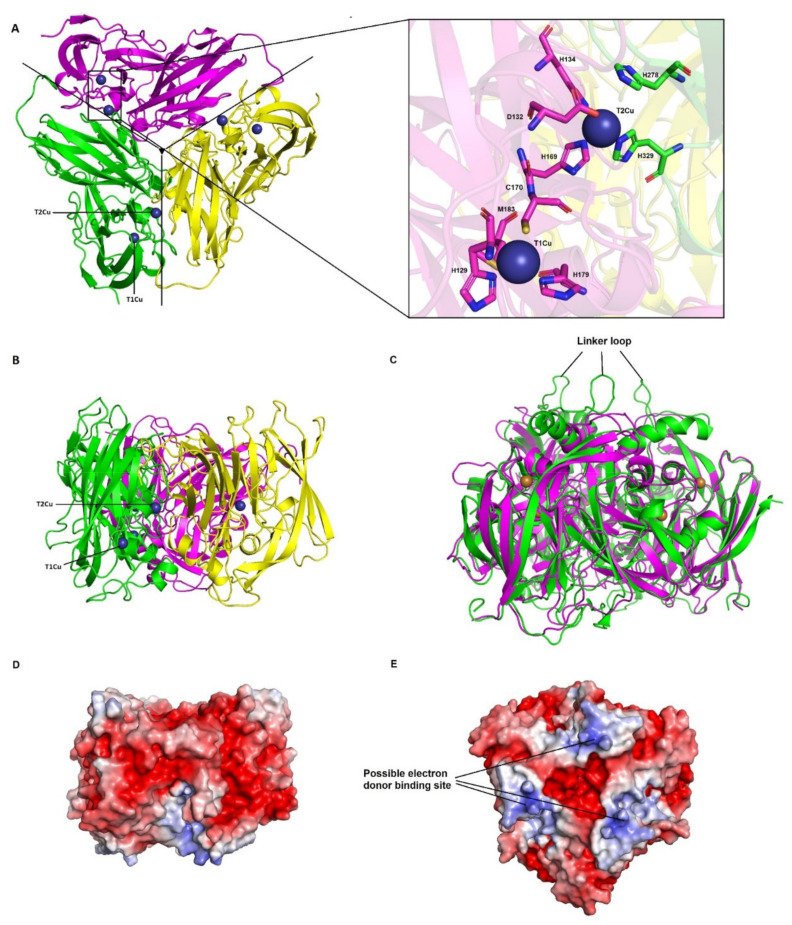
Homology model and electrostatic surface potential of *Haloferax mediterranei* NirK (**A**) Structural model of *Haloferax mediterranei* NirK in which the three subunits (in cartoon) are coloured in yellow, green, and magenta (**left**). The interface between monomers is indicated by a black continuous line. Cu atoms are represented as dark blue spheres and named T1Cu and T2Cu whereas amino acids involved in Cu coordination are shown in sticks (**right**). (**B**) 90° rotation view from A. (**C**) Superimposed structures of NirKs of *Alcaligenes xilosoxidans* (green; Uniprot: O68601) and *Haloferax mediterranei* (magenta; Protein NCBI ID: WP_004059594.1) highlighting linker loops. (**D**,**E**) Electrostatic surface potential of *Haloferax mediterranei* NirK trimer. D is in the same orientation as B whereas E is rotated 180° in vertical plane from A and 90° in horizontal plane from D. The electrostatic potential is represented between +5 and -5 *kT/e*.

A structural model of respiratory nitrite reductase (NirK) from haloarchaeon *Haloferax mediterranei* was obtained using the structure of the soluble domain of lipoprotein AniA from the pathogenic bacteria *Neisseria gonorrhoeae* (PDB: 5tb7.1A) as template. The sequence identity with the template was 44%, with a 93% of coverage and QMEAN and GMQE values of 0.64 and 0.76, respectively. AniA is a trimeric class II NirK with each monomer consisting of two β-sandwich cupredoxin domains [67,68]. As the template, the structural model of NirK from *Haloferax mediterranei* (Figure 6A,B) would be organized in three monomers constituting a homotrimer structure. Each monomer is composed by 327 amino acids with two cupredoxin domains to support copper atoms.

Clustal Omega alignment reveals that the most important amino acid residues involved in catalysis and electron transfer as well as copper-centre coordination are conserved in all analysed haloarchaeal species (Figure 5B and Figure S2). Furthermore, a SSFHV/I/P motif conserved around the active site has been found, being proline the last amino acid in most haloarchaeal genomes. This motif, involving the catalytic His residue, classifies haloarchaea NirKs into Clade II. This clade includes NirKs sequences belonging to Class II or unclassified, while Clade I comprises Class I enzymes [56,69]. Helen et al. (2016) observed that the conserved TRPHL and SSFHV/I/P motifs are not a reliable method of classification into Clade I or Clade II since this region is much more diverse than previously described. Nevertheless, enzymes belonging to Clade II have two deletion regions in two surface loops as common feature. The linker loop between the two cupredoxin domains is, in *Haloferax mediterranei* and in other NirK enzymes from Clade II, seven residues shorter than in Clade I enzymes (Figure 6C and Figure S3). In AniA, this shortened linker loop may promote a more intimate interaction between AniA and the surface of the outer membrane [67]. The second loop forms a large pedestal shaped tower structure. In Clade II enzymes the eight residue deletion results in a shortened α-helix and a refolded coil structure. This difference in the surface near the type I copper site could be important for binding of different electron donors [67].

The *Haloferax mediterranei* NirK model reveals the presence of the two copper sites with different coordination patterns. Firstly, type 1 copper site coordinated by two His residues (His_129_ and His_179_), one Cys (Cys_170_) and one Met (Met_183_). Secondly, type 2 copper centre is placed in the interface of two adjacent subunits, and it is coordinated by three His residues involving both subunits. His_134_ and His_169_ are provided by the N-terminal domain of one monomer and His_329_ by the C-terminal domain of the adjacent monomer (Figure 6A). Both copper ions are connected by His_169_, of the type 2 copper centre, and Cys_170_, of the type 1 (Figure S2). In well-studied NirK atomic resolution structures, type 2 copper centre has been reported to be coordinated by three His residues and one water molecule which are involved in the catalytic mechanism [70,71]. These coordination patterns (His_2_-Cys-Met and His_3_-H_2_O) have been detected and conserved in others two-domain NirK, three-domain NirK and four-domain NirK enzymes belonging to Bacteria and Archaea domains such as *Nitrosomonas europaea* [72], *Alcaligenes faecalis* [73], *Alcaligenes xylosoxidans* [74], *Ralstonia pickettii* [75], *Haloarcula marismortui* [19] or *Bradyrhizobium* sp. ORS 375 [62]. The catalytic mechanism of NirK involves Asp and His residues that donate two protons necessary for nitrite to nitric oxide conversion. These residues are conserved in *Haloferax mediterranei* NirK, being His_278_ and Asp_132_ (Figure 6A), and the proposed mechanism [70,76,77,78] could also take place in NirK from *Haloferax mediterranei*.

The physiological electron donors to NirK in *Haloferax mediterranei* is still unknown. It could be a halocyanin that it is encoded in the proximity of NirK [6]. In Figure 6D,E is represented the electrostatic potential of the trimer and possible areas of interaction with the electron donor can be identified.

### 3.3. Nitrous Oxide Reductase

The nitrous oxide reductase (Nos) catalyses the last step of denitrification. This enzyme has been extensively studied during the last twenty years in bacteria, particularly from soils, due to its relationship with climate change [79,80]. Nevertheless, there is scarce information about archaeal NosZ enzymes. To date, about ten Nos from different microorganisms have been isolated and none of them belong to the Archaea domain [81]. Phylogenetically, Nos are divided in two main groups: typical (Clade I) and atypical (Clade II) Nos [79,80,81]. In Clade I, Nos proteins are characterized by the presence of TAT signal peptides related to the TAT export system, whereas Clade II proteins use the Sec system for transport across membranes [81]. Another important difference is that some enzymes of Clade II also carry an additional cytochrome *c* domain at the C-terminal [81,82]. The Nos “core” structure is remarkably similar between clades, since both clades possess Cu_A_ and Cu_Z_ multicopper centres [79,80,81]. Cu_A_ is a binuclear centre, which is the electron transfer cluster, while Cu_Z_ is a tetranuclear cluster which is part of catalytic centre [81,83].

**Figure 7:**
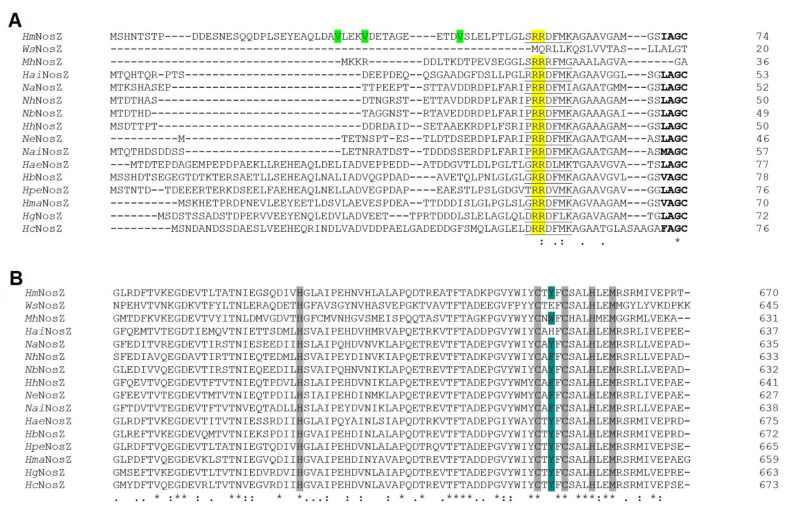
Clustal omega alignment of *Wolinella succinogenes* (*Ws*NosZ; Protein NCBI ID: WP_129545366.1), *Marinobacter hydrocarbonoclasticus* (*Mh*NosZ; Protein NCBI ID: WP_039373687.1), *Halorubrum aidingense* (*Hai*NirK; Protein NCBI ID: WP_007998714.1), *Natronococcus amylolyticus* (*Na*NirK; Protein NCBI ID: WP_049891949.1), *Natrialba hulunbeirensis* (*Nh*NosZ; Protein NCBI ID: ELY91737.1), *Natronolimnobius baerhuensis* (*Nb*NosZ; Protein NCBI ID: WP_087715262.1), *Halobiforma haloterrestris* (*Hh*NosZ; Protein NCBI ID: WP_089784324.1), *Natrinema ejinorense* (*Ne*NosZ; Protein NCBI ID: WP_097381668.1), *Natronorubrum aibiense* (*Nai*NosZ; Protein NCBI ID: WP_152944304.1), *Haloplanus aerogenes* (*Hae*NosZ; Protein NCBI ID: WP_121921888.1), *Haloferax mediterranei* (*Hm*NosZ; Protein NCBI ID: WP_004056356.1), *Halogeometricum borinquense* (*Hb*NosZ; Protein NCBI ID: WP_163487366.1), *Halolamina pelagica* (*Hpe*NosZ; Protein NCBI ID: SFP13007.1), *Haloarcula marismortui* (*Hma*NosZ; Protein NCBI ID: WP_011222995.1), *Hagranum gelatinilyticum* (*Hg*NosZ; Protein NCBI ID: WP_089699362.1), *Halosimplex carlsbadense* (*Hc*NosZ; Protein NCBI ID: WP_006884108.1). (**A**) N-terminal signal peptide region. The TAT consensus sequence [ST]RRxFLK is underlined, and lipoprotein consensus sequence is in bold. Twin arginine ‘RR’ and hypothetical start codons of *Haloferax mediterranei* NosZ are highlighted in yellow and green, respectively. (**B**) Cu_A_ centre coordination. Conserved residues coordinating Cu_A_ centre atoms are highlighted in grey whereas conservative substitutions are highlighted in turquoise.

*Haloferax mediterranei* nitrous oxide reductase lacks the characteristic *c*-haem binding motif and the Sec-dependent signal peptide, thus it belongs to the Clade I or “typical” NosZ (Figure 7A).

#### 3.3.1. N-Terminal Analysis Shows Same Annotation Conflict as in Respiratory Nitrate Reductase

*Haloferax mediterranei* NosZ harbours, as NirK, the TAT/lipobox signal peptide, pointing out the enzyme could be anchored to the membrane through Cys_74_. The signal peptide is larger than typical ones and the consensus motif is far from the N-terminus (Figure 7A). This feature could indicate, as in NarG, that the NosZ ORF may not be well annotated, and its start could be present closer to the consensus motif. Three probable ORF starts are Val_30_, Val_34_ or Val_44_, being GUG the start codon (Figure 7A and Figure S4).

#### 3.3.2. Cu_A_ and Cu_Z_ Centres Are Conserved in Haloarchaea

**Figure 8:**
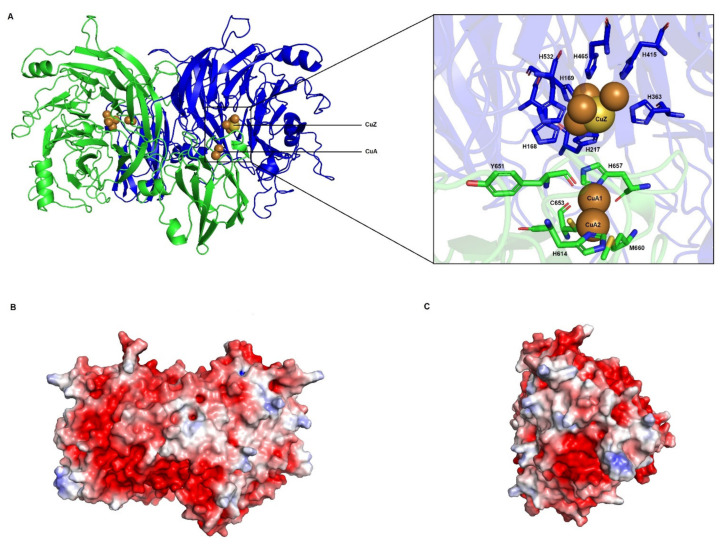
Homology model and electrostatic surface potential of Haloferax mediterranei NosZ (**A**) Three-dimensional model of NosZ in which two subunits (in cartoon) are coloured in green and blue (**left**). CuA centre is represented as two brown spheres whereas CuZ centre is represented as yellow sphere surrounded by four brown spheres. Amino acids involved in Cu centres coordination are shown in sticks (**right**). (**B**,**C**) Electrostatic surface potential of Haloferax mediterranei NosZ dimer. The electrostatic potential is represented between +5 and -5 kT/e.The image B is in the same position that image A and the image C is rotated 90º with respect to image B.

The *Haloferax mediterranei* NosZ structural model (Figure 8) has been conducted using as template the crystal structures of NosZ from *Marinobacter hydrocarbonoclasticus* (*Pseudomonas nautica*) (PDB: 1qni.1) [84]. The sequence identity was 53%, with a coverage of 96% and a QMEAN of -2.74 and GQME of 0.69. Although GQME was higher using as template *Pseudomonas stutzeri* (PDB: 6rkz.1, 49% sequence identity, 96% of coverage and QMEAN and GMQE values of -2.09 and 0.81, respectively) we decided to use *Marinobacter hydrocarbonoclasticus* enzyme for modelling *Haloferax mediterranei* NosZ, since there was low score differences between models, and *Marinobacter hydrocarbonoclasticus* is an extreme halotolerant microorganism [85]. This bacterium was considered a better fit for this modelling study given that proteins adapted to salty environments share features among them, such as the presence of acidic residues [86]. In comparing amino acid composition between template and query we found that the *Marinobacter hydrocarbonoclasticus* enzyme sequence is composed of approx. 8.6% Asp and 5.3% Glu, whereas percentages for *Haloferax mediterranei* NosZ are 9.8% and 7.3%, respectively. Furthermore, theoretical pI of both enzymes has been determined bioinformatically at 4.7. These values point toward the fact that these enzymes are acidic and reinforce the choice of *Marinobacter hydrocarbonoclasticus* NosZ as the most suitable template for this study. Template and query show good similarity and superimposing, even considering that NosZ sequence from *Haloferax mediterranei* is larger than *Marinobacter hydrocarbonoclasticus*. Structural modelling suggests that *Haloferax mediterranei* NosZ is, like the template, a homodimer [84]. Each monomer presents two domains that bind the copper centres Cu_A_ and Cu_Z_. Cu_A_ is a binuclear copper centre located at the C-terminal domain of the enzyme whose main function is the electron transport from small electron carrier proteins to the tetranuclear copper sulphide catalytic centre Cu_Z_ situated at the N-terminal [81,84].

Cu_A_ centre is coordinated by a cupredoxin fold organized in nine β-strands. Structure and coordination of this binuclear centre are well studied in *Marinobacter hydrocarbonoclasticus* NosZ crystal structure [84]. This coordination is carried out mainly by six residues: Cys_611_ and Cys_615_ which bind the copper atoms; His_576_ and Met_622_ that coordinate one of the Cu_A_ centre (Cu_A1_) while Trp_613_ and His_619_ bind Cu_A2_ centre [84].

These residues seem to be conserved (or partially conserved) among species, including *Haloferax mediterranei* and other haloarchaea B and Figure S4) [87]. The Trp that coordinates Cu_A2_ centre is replaced by another aromatic residue (Tyr or Phe) in almost all the haloarchaea, being it a conservative replacement. The only exception is *Halorubrum aidingense*, in which Trp is replaced by His.

**Figure 9:**
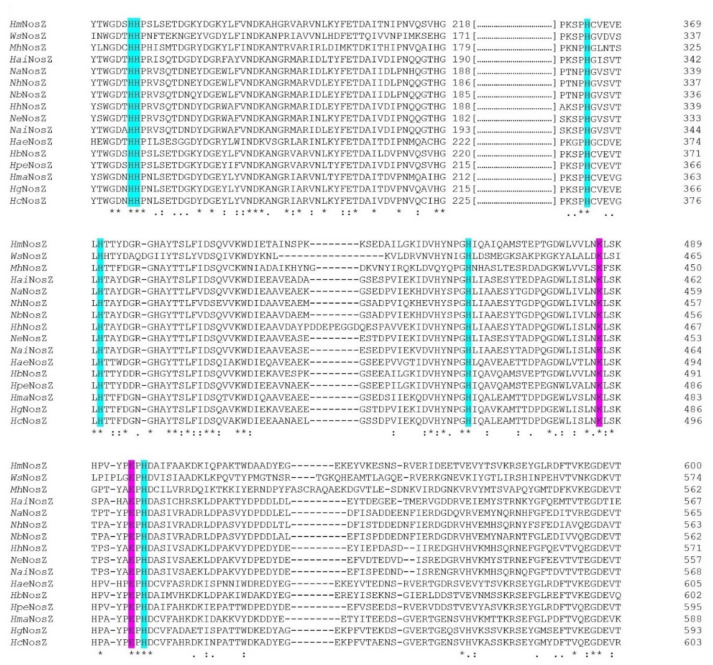
Clustal omega alignment of the Cu_A_ centre coordination region from *Wolinella succinogenes* (*Ws*NosZ; Protein NCBI ID: WP_129545366.1), *Marinobacter hydrocarbonoclasticus* (*Mh*NosZ; Protein NCBI ID: WP_039373687.1), *Halorubrum aidingense* (*Hai*NirK; Protein NCBI ID: WP_007998714.1), *Natronococcus amylolyticus* (*Na*NirK; Protein NCBI ID: WP_049891949.1), *Natrialba hulunbeirensis* (*Nh*NosZ; Protein NCBI ID: ELY91737.1), *Natronolimnobius baerhuensis* (*Nb*NosZ; Protein NCBI ID: WP_087715262.1), *Halobiforma haloterrestris* (*Hh*NosZ; Protein NCBI ID: WP_089784324.1), *Natrinema ejinorense* (*Ne*NosZ; Protein NCBI ID: WP_097381668.1), *Natronorubrum aibiense* (*Nai*NosZ; Protein NCBI ID: WP_152944304.1), *Haloplanus aerogenes* (*Hae*NosZ; Protein NCBI ID: WP_121921888.1), *Haloferax mediterranei* (*Hm*NosZ; Protein NCBI ID: WP_004056356.1), *Halogeometricum borinquense* (*Hb*NosZ; Protein NCBI ID: WP_163487366.1), *Halolamina pelagica* (*Hpe*NosZ; Protein NCBI ID: SFP13007.1), *Haloarcula marismortui* (*Hma*NosZ; Protein NCBI ID: WP_011222995.1), *Hagranum gelatinilyticum* (*Hg*NosZ; Protein NCBI ID: WP_089699362.1), *Halosimplex carlsbadense* (*Hc*NosZ; Protein NCBI ID: WP_006884108.1). Conserved His residues coordinating Cu_Z_ centre are highlighted in cyan and conserved Lys and Glu are in magenta.

Cu_Z_, is located at the N-terminal and has a seven-bladed-β-propeller fold. This center has been only identified in NosZ enzymes and contains four sulphide bridged copper atoms coordinated by seven His residues [81,84]. In the case of *Marinobacter hydrocarbonoclasticus* NosZ (and other NosZ), each copper is coordinated by two His except from CuIV atom which is coordinated by only one. His residues involved in the coordination of copper atoms in *Marinobacter hydrocarbonoclasticus* are: His_129_, His_130_, His_178_, His_320_, His_375_, His_426_ and His_487_ (Figure 9A and Figure S4). Cu_Z4_ can attach an oxygen atom from a water molecule or a hydroxide, which is the proposed substrate binding position [84,88,89]. This arrangement receives the name of 4Cu1S or simply Cu_Z_* centre [81]. Apart from those His residues, there are two important amino acids related to the stabilization of the Cu_Z_* centre, Lys_447_ and Glu_485_. Lys_447_ seems to establish a hydrogen bond with a hydroxide ligand which is bound to Cu_Z4_ and another hydrogen bond with Glu_485_ [90,91,92].

On the one hand, sequence alignments display that key coordination residues are conserved among haloarchaea species (Figure 9). The seven His are present together with the Lys and Glu residues. On the other hand, the model maintains its characteristic distorted tetrahedron geometry (Figure 8A). These data provide evidence to support that the Cu_Z_ centre is well conserved in *Haloferax mediterranei* and in other haloarchaea and suggest that the catalytic reaction of nitrous oxide reduction to dinitrogen happens in the same way as in *Marinobacter hydrocarbonoclasticus*.

#### 3.3.3. Electron Transfer Pathways Show Partial Conservation and Similarity to Clade II NosZ

Electron transfer in this enzyme encompasses two steps, electron transfer from a small electron donor to the Cu_A_ centre and electronic transfer from Cu_A_ to Cu_Z_ centre. Using molecular docking approaches, Dell’Acqua and coworkers have proposed different possible conserved electron routes in *Marinobacter hydrocarbonoclasticus* among other microorganisms [83]. Electrons from small molecules are transferred to the Cu_A_ centre. In the case of *Marinobacter hydrocarbonoclasticus*, the amino acids which are involved in this process are: H_616_ (which is supposed to be the entry point of electrons), Leu_618_, Asp_569_, Ala_545_, Val_574_ and Gln_547_ (Figure S4) [83,87]. Then, electrons from Cu_A_ must be transferred to Cu_Z_ centre to achieve a fully Cu_Z_ centre reduction (4Cu^+^), which is the most favourable state for N_2_O binding [83,93]. The most probable hypothesis indicates that electrons are transferred firstly to the Trp_613_, subsequently to Phe_614_ and finally to the oxygen of the water molecule or hydroxyl group bound to Cu_Z4_ (Figure S4). In the other alternative pathway, the electron transference starts at the His_619_, follows at Met_620_, and then it finishes at His_178_ which is a ligand of the Cu_Z2_ (Figure S4) [83].

Combining these data with sequence alignment, potential presence of this electron pathway has been analysed, not only in *Haloferax mediterranei* NosZ but also in other haloarchaea. Thus, considering the residues implicated in the first step of the electron transport pathway in *Marinobacter hydrocarbonoclasticus*, it is concluded that this pathway is not conserved in haloarchaea. Surprisingly, some haloarchaeal residues in these positions are more similar to those in *Wolinella succinogenes* NosZ, which belong to the Clade II of NosZ. These residues, Ala_545_, Asp_569_ and His_616_, are replaced by Arg, Glu, and Ser, respectively (Gln_529_ and Val_524_ are residues with a certain variability between species) [83]. Furthermore, Arg_557_ and Cys_627_ have been proposed to carry out electron transport pathway between *c*-haem-containing domain and Cu_A_ in *Wolinella succinogenes* [83]. In fact, these residues are well conserved in haloarchaea. Nevertheless, haloarchaeal NosZ lack *c*-haem-containing domains. Figure 8B,C shows the possible electron binding site on *Haloferax mediterranei* NosZ. Transport from Cu_A_ to Cu_Z_ is more conserved. The two proposed pathways are possible because all mentioned residues are conservatively replaced. The most probable route only changes Trp_613_ for another aromatic residue (except from *Halorubrum aidingense*), whereas the alternative route replaces Met_620_ by Leu.

## 4. Conclusions

Sequence analysis of the main denitrification enzymes indicates that they are associated with the plasma membrane on the extracellular side. The conserved lipobox in NirK and NosZ point out that both could be membrane-bound lipoproteins that are exported through a TAT system. Although it is not known with certainty which is the electron donor protein to NarH and how it is associated with the plasma membrane. Even though the orientation in the membrane is reverse, the residues involved in cofactors coordination and in the electron transfer, are highly conserved in haloarchaeal nitrate reductases. Catalytic mechanism and electron transfer in NirK and NosZ are likely remarkably similar to bacteria, as well. The structural models of the enzymes make possible the identification of key residues involved in the discrimination of substrates, electron transfer or catalytic activities allowing the design of directed mutagenesis experiments to confirm the role of these residues. As nitrate reductase from *Haloferax mediterranei* binds its substrate from the extracellular media, where is expected to be more diluted than in cytoplasm, the substrate affinity must be higher when comparing with bacterial nitrate reductase, as occurs in the comparation with *Escherichia coli* nitrate reductase. Directed mutagenesis experiments will be needed to confirm the role of the residues assigned to substrate binding and responsible for the binding affinity. Nevertheless, further structural, and spectroscopic analyses are required to confirm the mechanisms responsible for *Haloferax mediterranei* denitrification to identify the intermediate electron donors and acceptors and to describe the bioenergetics associated with this respiratory pathway configuration. Clearly, the description of haloarchaeal denitrification remains in their infancy compared to denitrification pathways in bacterial model. Importantly, there is a notable lack of crystallographic structural data for haloarchaeal denitrification enzymes due to overexpression problems and low protein stabilization. For this reason, the optimization of the overexpression systems for haloarchaeal protein is necessary. These methodological improvements could increase the number of solved haloarchaeal crystallographic structures; therefore, shedding some light on the electron pathways and catalytic mechanism involved. In conclusion, our results set the basis for the understanding of the molecular mechanism involved in denitrification, which will help in the optimization of the use of these microorganisms in bioremediation of wastewater and brines in the near future.

## Figures and Tables

**Table 1:** Michaelis constant (K_m_, mM) comparison for *Ao*PcrAB-Wild type and *Ao*PcrAB-PcrA W489E from *Azospira oryzae*, *Ec*NarGHI from *Escherichia coli* and *Hm*NarGH from *Haloferax mediterranei*. N.S means not a substrate.

	Perchlorate	Chlorate	Bromate	Iodate	Nitrate	References
***Ao*** **PcrAB-Wild type**	6.0 ± 2.1	7.4 ± 2.1	4.4 ± 3.8	11 ± 5.4	23 ± 5.2	[35]
***Ao*** **PcrAB- PcrA W489E**	301 ± 14.1	20.5 ± 3.2	4.8 ± 2.1	1.27 × 10^5^ ± 3.5 × 10^4^	1.59 × 10^4^ ± 4.5 × 10^3^	[35]
***Ec*** **NarGHI**	1060 ± 154	113 ± 35	2690 ± 502	N.S	202 ± 65	[35,52]
***Hm*** **NarGH**	Unquantified detected activity	2.41 ± 0.16	Unquantified detected activity	N.S	0.82 ± 0.14	[20,51]

## Supplementary Materials

The following are available online at https://www.mdpi.com/article/10.3390/biom11071043/s1, Figure S1: Complete alignment of NarG sequences of bacteria and haloarchaeal species included in this study, Figure S2: Complete alignment of NirK sequences of bacteria and haloarchaeal species included in this study, Figure S3: Complete alignment of NirK sequences of Clade I and Clade II species included in this study, Figure S4: Complete alignment of NosZ sequencesof bacteria and haloarchaeal species included in this study, Table S1: Interaction distance (Angstroms) between the enzyme residues and the metal centres in NarG, NirK and NosZ templates and models.
