# Supplementary material for: In Silico Analysis of the Enzymes Involved in Haloarchaeal Denitrification
Source: Biomolecules. 2021 Jul 16;11(7):1043. doi: 10.3390/biom11071043 (PMC8301774; doi:10.3390/biom11071043)
Supplement: Supplementary file 1 [file biomolecules-11-01043-s001.zip › biomolecules-1267674-supplementary.pdf]

Article

In Silico Analysis of the Enzymes Involved in Haloarchaeal Denitrification

Eric Bernabeu[1], Jose María Miralles-Robledillo[1], Micaela Giani[1], Elena Valdés[1], Rosa María Martínez-Espinosa[1,2], Carmen Pire[1,2*]

[1] Biochemistry and Molecular Biology Division, Agrochemistry and Biochemistry Department, Faculty of Sciences, University of Alicante, Ap. 99, E-03080 Alicante, Spain
[2] Multidisciplinary Institute for Environmental Studies "Ramón Margalef" University of Alicante, Ap. 99, E-03080 Alicante, Spain
* Correspondence: carmen.pire@ua.es

Supplementary Material

```
HmNarG     1 MSRNDASQLDDGETTA------E-SPPDDQANDAPEVGD-----PPGDPVDADSGVSRRTFLEGIGVASLLGIGTSAA-S
HmaNarG    1 ---------------------------------------MSRNDLTDD-EGDSAGISRRDFVRGLGAASLLGAT----GL
HlNarG     1 MSD-----------------------------------SHDF-HDDSESTTGGIDAARRDFLKGIGLGAVLGLGGVSVA-
HsNarG     1 MS------------------------------------DNQSNVDVENADESNSGVGRRGFLKGLGLTTALGLTGTTIG-
HpNarG     1 MTRNDSRDRTTDA--S------T-DERTDGGAGGPDVGD-----PPGNPSSA--RLSRRGFLEGVGLASILGIGGASA-T
HaNarG     1 MTD---------------------------------RHEATE---RDESNTLLSGVDRRGFLKGIGVAGLLGASGTGLA-
HmuNarG    1 ---------------------------------------MSEHDIDDDRWMDSSGITRRDFVRGLGAASIVGAT----GL
HuNarG     1 ------------------------------------------MSDPTQSDDSTDGVSRRDFLLGAGAAGVVGATGLTVA-
HrNarG     1 MSE--------------------------------------RHDTT--DESDGIEAARRDFLKGLGAATAVGVTGFGLG-
HliNarG    1 MTD--------------------------------------TTDASDESTRDGSGAARRDFLKGVGAAAAVGATGLGSA-
HcNarG     1 MTDDHSTDLTDDPPTDSTDSEATDSDTDVSTDGVPASGASGGSSELRSDGGAGVDVSRRDFLKGVGAAGVAGAGASGTGL
SsNarG     1 MSE---------------------------------RENENYRDSTSASATGDVSTARRDFLKGIGVASLLGATGTGLGA
AoPcrA     1 ----------------------------------------------------MVQMTRRGFLLASGATLL-GS-------
EcNarG     1 --------------------------------------------------------------------------------
SeNarG     1 --------------------------------------------------------------------------------
BcNarG     1 ----------------------------------------------------------------------MK------KK
BfNarG     1 MTI---------------------------------------Q-----DRHASPGRSAQPAPEGTGSPGIDS------PL
BpNarG     1 --------------------------------------------------------------------------------

HmNarG    68 D----DSLFQM-GGLKPVD--DPIGNYPYRDWEDLYREKWDWDSVSRSTHSVNCTGSCSWNVYVKNGQVWREEQSGDYPR
HmaNarG   37 S----FADDGM-DGLEAVD--DPIGSYPYRDWEDLYRDEWDWDSVARSTHSVNCTGSCSWNVYVKDGQVWREEQAGDYPT
HlNarG    44 -----DRQLDM-DGLEVVD--DPIGSYAYRDWEDLYREEWDWDSTARSTHSVNCTGSCSWEVYVRNGQVWRESQAGDYPQ
HsNarG    44 -----DELTQM-GGIENVDGSDYIGSYPYREWEDFYREKWDWDSVARSTHGVNCTGSCSWNVYVKNGQVWREEQAADYPE
HpNarG    64 G----ETLFSM-DGLRPVD--DPIGEYPYREWEDLYREEWDWDSVARSTHSVNCTGSCSWNVYVKNGQVWREEQAGDYPR
HaNarG    44 -----DDFLRM-DGLEVVD--DPIGNYPYRDWEDLYREEWDWDSTARSTHSVNCTGSCSWEVFVRNGQVWRESQAGDYPR
HmuNarG   38 S----FADEEM-DGLQAVD--DPIGSYPYREWEDLYREEWDWDSVARSTHSVNCTGSCSWNVYVKDGQVWREEQAGDYPV
HuNarG    38 -------DRAL-DGLETVD--DPIGNYPYRDWEDFYREEWDWDSVARSTHSVNCTGSCSWDVYVKNGQVWREQQANDYPT
HrNarG    40 -----QQ-SEM-QSLEIVD--DPIGSYPYRDWEDLYREEWDWDSKARSTHSVNCTGSCSWQVYVRNGQVWREEQAGDYPR
HliNarG   42 -----QDLTEM-TTLEVVD--DPIGDYPYRDWEDLYREEWDWDGKARSTHSVNCTGSCSWQVYTRNGQVWREEQAGDYPR
HcNarG    81 A----QDFLQM-TRLQVVN--DPIGDYPYREWEDLYREEWDWDSKHRSTHSVNCTGSCSWNVYVKNGQVWREEQAGDYPN
SsNarG    48 TGDELDEFFQL-DGIEVVD--DPIGDYPYRDWEDLYREQWDWDSTARSTHSVNCTGSCSWEVYVRNGQVWREDQAADYPV
AoPcrA    21 -------SLSL-RTLA--AATDISGAFEYSGWENFHRTQWSWDKKTRGAHLVNCTGACPHFVYSKDGVVMREEQSKDIAP
EcNarG     1 MSKFLDRFRYFKQKGETFADGHGQLLNTNRDWEDGYRQRWQHDKIVRSTHGVNCTGSCSWKIYVKNGLVTWETQQTDYPR
SeNarG     1 --MGKFGLNFFKP-TEKFNGNWSVLEHKSREWEKMYRERWSHEKVVRTTHGVNCTGSCSWKVFVKNGVITWENQQIDYPS
BcNarG     5 PSALMRRLKYFSP-IDRYNDNHTQETYEDREWENVYRKRWQHDKVIRSTHGVNCTGSCSWNIYVKDGIVTWEGQELNYPT
BfNarG    31 FDALIGARRMFSRSERISEDNRETHKVGGRSGDSFYRERWSHDKVVRSTHGVNCTGSCSWKVYVKDGVITWESQETDYPS
BpNarG     1 MSHFLDRLKFMSRVKSTFSDGHGAVVDEDRRWENGYRSRWQHDKIVRSTHGVNCTGSCSWKVYVKNGLITWETQQTDYPR
```

```
HmNarG    141 FDESLPDPNPRGCQKGACYTDYVNADQRIKHPLKRV-------------------------------------GERGEGKW
HmaNarG   110 FDESLPDPNPRGCQKGACYTDYVNADQRVLHPLRRT-------------------------------------GERGEGQW
HlNarG    116 FDESLPDPNPRGCQKGACYSDYVNADHRVLHPLRRT-------------------------------------GERGEGMW
HsNarG    118 INDELPSSNPRGCQKGACYTDYVNAEQRITHPLRRV-------------------------------------GERGEGKW
HpNarG    137 FDESVPDPNPRGCQKGACYTDYVNADERIKHPLKRV-------------------------------------GERGEGKW
HaNarG    116 FDESVPDPNPRGCQKGACYSDYVNADQRIKYPLRRT-------------------------------------GARGEGKW
HmuNarG   111 IDEDLPDPNPRGCQKGACYTDYVNADQRVLHPLRRT-------------------------------------GERGEGQW
HuNarG    108 FDESLPDPNPRGCQKGACYNDYVDAEQRVQYPMRRT-------------------------------------GERGAGEW
HrNarG    111 FDESLPDPNPRGCQKGACYSDYVNADQRILHPLKRV-------------------------------------GERGEGQW
HliNarG   114 FDESLPDPNPRGCQKGACFTDYVNADQRVTHPLRRT-------------------------------------GERGEGKW
HcNarG    154 FDESLPDPNPRGCQKGACYTDYVNADQRIKYPMRRT-------------------------------------GERGEGKW
SsNarG    125 FDESLPNPNPRGCQKGACYSDYVNADHRVLHPLRRT-------------------------------------GERGSGKW
AoPcrA     91 M-PNIPEYNPRGCNKGECGHDYMYGPHRIKYPLIRV-------------------------------------GERGEGKW
EcNarG     81 TRPDLPNHEPRGCPRGASYSWYLYSANRLKYPMMRKRLMKMWREAKAL-H-SDPVEAWASIIEDADKAKSFKQARGRGGF
SeNarG     78 CGPDMPEFEPRGCPRGASFSWYEYSPLRVKYPYIRGKLLDLWTEALEE-QKGNRIAAWASIVENEEKAKQYKEARGKGGH
BcNarG     84 TGPDMPDFEPRGCPRGASFSWYIYSPLRVKYPYVRGVLWDMWQEELQN-N-ESPLGAWKSIVENPEKARTYKQARGKGGF
BfNarG    111 TGPDKPEYEPRGCPRGASFSWYTYSPTRVRYPYVRSELLRMFREEKAKAPGQDPVEAWKAIVSDPEKAMRYKRARGKGGL
BpNarG     81 TRADLPNHEPRGCPRGASYSWYVYSAQRVKYPMIRGRLMQMWREARK--T-MDPIAAWESISQNPEKARRYKSVRGLGGF

HmNarG    185 KRISWDEALTEIAEHVVDEVE-AGRYDAISGFTPIPAMSPVSFASGSRLVNLLGGVSHSFYDWYSDLPPGQPITWGTQTD
HmaNarG   154 ERISWDEALTEIADHVIDEVQ-AGRYDAISGFTPIPAMSPVSFASGSRLVNLLGGVSHSFYDWYSDLPPGQPITWGTQTD
HlNarG    160 ERISWDEALTEIAEEVVDTVR-AGEYDAISGFTPIPAMSPVSFASGSRLINLLGGVSHSFYDWYSDLPPGQPITWGTQTD
HsNarG    162 KRISWDEALTEIAEKVIEEVQ-QGNYDAISGFTPIPAMSPVSFASGSRFVNLIGGVSHSFYDWYSDLPPGEPITWGTQTD
HpNarG    181 QRISWDEALTEIAEHVVGEVQ-AGRYDAISGFTPIPAMSPVSFASGSRLINLLGGVSHSFYDWYSDLPPGQPITWGTQTD
HaNarG    160 QRISWDEALTEIAEKLVEEVT-AGRYDAISGFTPIPAMSPVSFASGSRLVNLLGGVSHSFYDWYSDLPPGQPITWGHQTD
HmuNarG   155 ERISWDEALTEIADHVIDEVQ-AGRYDAISGFTPIPAMSPVSFASGSRLVNLLGGVSHSFYDWYSDLPPGQPITWGTQTD
HuNarG    152 ERISWDEALTEIAEHVIEEIQ-NGRYDAISGFTPIPAMSPVSFASGTRLVNLLGGVSHSFYDWYSDLPPGQPITWGHQTD
HrNarG    155 QRISWDEALTEIAEEVIDTVQ-AEEYDGISGFTPIPAMSPVSFASGSRLVNLLGGVSHSFYDWYSDLPPGQPITWGTQTE
HliNarG   158 ERISWDEALTEIAEEVIDAVE-DEEYDAISGFTPIPAMSPVSFASGSRLINLLGGVSHSFYDWYSDLPPGQPITWGTQTD
HcNarG    198 QRISWDEALTEIAEHVVDEVE-AGRYDAISGFTPIPAMSPVSFASGSRLVNLLGGVSHSFYDWYSDLPPGQPITWGTQTD
SsNarG    169 ERISWDEALTEIAEEVIETVAEEENYDGISGFTPIPAMSPVSFAGGSRLVNLLGGVSHSFYDWYSDLPPGQPITWGTQTD
AoPcrA    134 RRATWEEALDMIADKCVDTIK-NHAPDCISVYSPVPAVSPVSFSAGHRFAHYIGAHAHTFYDWYGDHPTGQTQTCGVQGD
EcNarG    159 VRSSWQEVNELIAASNVYTIK-NYGPDRVAGFSPIPAMSMVSYASGARYLSLIGGTCLSFYDWYCDLPPASPQTWGEQTD
SeNarG    157 VRANWKDATDIIAAQILYTIK-KDGPDRIAGFTPIPAMSMISYASGARFINLLGGEMLSFYDWYADLPPASPQIWGEQTD
BcNarG    162 IRANWDEVLQLVSASLLYTVL-KYGPDRNVGFSPIPAMSMLSHAAGSRFMQLMGGPMLSFYDWYADLPPASPQIWGDQTD
BfNarG    191 VRASWDEAVELVAAAYVHTVK-EHGPDRATGFSPIPAMSQVSFSSGARFHQLIGSSMLSFYDWYADLPPASPQVFGDQTD
BpNarG    158 VRADWNTATEIIAAANAYTIK-RYGPDRVVGFSPIPAMSMVSYAAGARYLSLIGGACLSFYDWYCDLPPASPQVWGEQTD

HmNarG    264 NAESADWYNADYIIAWGSNINVTRIPDAKYFLESGYNGTKRVGVFTDYSQTAIHTDEWLSPDSGTDTALALGMAQTIVDE
HmaNarG   233 NAESADWHNADYIIAWGSNINVTRIPDAKYFLDAGYEGAKRVGIFTDYSQTAIHTDEWLSPHGGSDTALALGMAQTIVDE
HlNarG    239 NAESADWYNADYIIAWGSNVNVTRIPDAKYFLEAAYNGTKRVGVFTDYSQTAIHCDEWLSPEPGTDTALALGMARTIVDE
HsNarG    241 NAESADWYDADYIIAWGSNINVTRIPDAKFFLEAAYKGTKRVGIFTDYSQTAIHCDDWLSPKAGSDAALALGMARTIVDE
HpNarG    260 NAESADWYNADYIIAWGSNINVTRIPDAKYFLEAGYNGTKRVGVFTDYSQTAIHTDEWLSPEPGADTALALGMAQTIVSE
HaNarG    239 NAESADWYNADYIVAWGSNINVTRIPDAKYFLEAGYNGTKRVGVFTDYSQTAIHCDEWIGPTPGTDTVLALGMARTIVDE
HmuNarG   234 NAESADWHNADYIIAWGSNINVTRIPDAKYFLDAGYEGAKRVGIFTDYSQTAIHTDEWLAPEGGTDTALALGMAQTIVDE
HuNarG    231 NAESADWHNAEYIIAWGSNINVTRIPDAKYFLDAGYEGAKRVGVFSDYSQTAIHTDEWIAPDPGTDTALALGMARTIVEE
HrNarG    234 NAESADWYNSDYIIAWGSNINVTRIPDAKYFLEAAYNGTKRVGIFTDYSQTAIHTDEWISPEPGTDTALALGMARVIVDE
HliNarG   237 NAESADWYNADYIIAWGSNINVTRIPDAKYFLEAGYDGAKRVGIFTDYSQTAIHCDEWLGPEPGSDTALALGMARTIVDE
HcNarG    277 NAESADWYNADYIIAWGSNINVTRIPDAKFFLDAAYNGTKRVGVFTDYSQTAIHTDEWLSPEGGTDTALALGMAQTIVDE
SsNarG    249 NAESADWYNADYLIAWGSNVNVTRIPDAKYFLEAAYDGTKRVGVFTDYSQTAIHCDEWISPEPGTDTALALGMARTIVDE
AoPcrA    213 TCETADWFNSKYIILWGSNPTQTRIPDAHFLSEAQLNGAKIVSISPDYNSSTIKVDKWIHPQPGTDGALAMAMAHVIIKE
EcNarG    238 VPESADWYNSSYIIAWGSNVPQTRTPDAHFFTEVRYKGTKTVAVTPDYAEIAKLCDLWLAPKQGTDAAMALAMGHVMLRE
SeNarG    236 VPESSDWYNASYIMMWGSNVPLTRTPDAHFMTEVRYKGAKVISVAPDYAENVKFADHWLAPHPGTDAAVAQAMTHVILQE
BcNarG    241 VPESSDWYNSGYIMTWGSNVPMTRTPDAHFLAEVRYKGTKVVSVSPDFAESTKFADDWISVKQGTDGALAMAMGHVILQE
BfNarG    270 VPESGDWWNASYLMMWGSNVPLTRTPDAHWMAEARYRGQKVISIAPDYAENVKFADEWLAPHPGTDAALAMAMGHTILRE
BpNarG    237 VPESADWYNSSYLLVWGSNVPQTRTPDAHFYTEVRYKGTKTVAISSDYGEMVKFGDIWLAPKQGTDAALAMAMGHVVLKE

HmNarG    344 GLYD------EAHLKEQTDMPLLVRQD--------TGKFLRASDVPSVNT---DADRPEW-MLLMLDSNGRIREAPGSLG
HmaNarG   313 GLHD------EAHLKEQTDMPLLVRED--------TGKFLRASEVGL-AE---DADDPEK-VFVMVDADGTLRRAPGSLG
HlNarG    319 GLHD------EAHLKEQTDMPLLVRED--------TGKFLRASEVSGVGG---GADDPEK-VFVMRDASGNLRAAPGSLG
HsNarG    321 GLHD------EAHLKEQSDMPFLVRED--------TGKFLRVADVPSLST---S-GEAGK-VFAMVDQQGNLRNAPGSLG
HpNarG    340 DLYD------EAHLKEQTDMPLLVRQD--------TGKFLRASDVPSVDT---DADRPEW-MLLMLDSEGSLREAPGSLG
HaNarG    319 ELYD------EAHLKEQTDMPLLVRED--------TGKFLRSADIRD--------GGDEN-VFVMQDADGTLRTAPGSLG
HmuNarG   314 ELYD------ESHLKEQTDMPLLVRED--------TGKFLRAGEVGL-AA---DADDPDK-VFVMVDEDGELRPAPGSLG
HuNarG    311 ELYD------EAHLKEQSDMPLLVRND--------TGKFLRASEVPGLSV---AADEPEK-VMVMQDGEGNLRAAPGSLG
HrNarG    314 GLYD------EAHLKEQTDMPLLVRAD--------TGKFLRVSDVPGLTT---GADRPDQ-MFVMQDGQGNLRVAPGSLG
HliNarG   317 GLYD------EAHLKEQTDMPLLVRED--------TGKFLRASEVSGLDV---DADRPEK-VFVMQDADGALRPAPGSLG
```

```
HcNarG   357 DLHD------EAHLKEQTDMPLLVRED--------TGKFLRASEVPSVGD---DVDRPEK-TFVMVDSEGSLRMAPGSLG
SsNarG   329 GLYD------EPHLKEQTDMPLLVRED--------TGKFLRASEI-GLDV---DADRPDH-VFVMQDGAGELRAAPGSLG
AoPcrA   293 KLYD------AHSLKEQTDLSYLVRSD--------TKRFLREADVVAGG-------SKDK-FYFWNAKTGKPVIPKGSWG
EcNarG   318 FHLDNPSQYFTDYVRRYTDMPMLVMLEERD-GYYAAGRMLRAADLVDALG---QENNPEWKTVAFN-TNGEMVAPNGSIG
SeNarG   316 YYENQPNDMFINYAKQYSDMPFVIMLDEDE-NGYKAGRFLRASDLGMS------GENNEWKPVIQDKLSQQLLVPNGTMG
BcNarG   321 FYVDNQVEYFTKYAKQYTDFPFFVTLKQKG-DQFVADRFLNAADIGRE------TKLGEWKPVLWNENTNDFATPHGTMG
BfNarG   350 FYVDRQEPYFESYSTQYTDLPFLVQLEQRDDGSLVPGKFLVASEAGTAITDEAETEHADFKPMLFDTATQAPAVPGGTLG
BpNarG   317 FHASNQSAYFRDYVKQYTDMPMLVMLRERD-GALVPDHFLRASHLAASLS---EANHPEWKTLAIDAATGDIVAPNGSIG

HmNarG   406 ERDGQKDYS-----------------------------------------KSI-------ELDFDPQL---DGETTVQTQ
HmaNarG  374 ERDGQKDYS-----------------------------------------ASI-------ELDFDPQL---SVERSVDTD
HlNarG   381 DRDGQHDAS-----------------------------------------ASI-------ELGFDPQL---SAEGSVSTT
HsNarG   382 NRDGKHDDS-----------------------------------------ASI-------ALDFDPRL---DVNQSVDLQ
HpNarG   402 ERDGQKDYS-----------------------------------------KSI-------ELDFDPRL---GAETTVQTT
HaNarG   376 NRAGKYDSE-----------------------------------------ASI-------ELEFDPQL---AVDRTVSTG
HmuNarG  375 ERDGQHDPE-----------------------------------------SSI-------ELDFDPQL---GVERSVETD
HuNarG   373 EREAKYDDS-----------------------------------------LSI-------ELDFDPQL---AVEDTVGTT
HrNarG   376 ERDGQYDPS-----------------------------------------ESI-------SLNFDPQL---AVERSVQTT
HliNarG  379 ERDGQYDDS-----------------------------------------VSI-------ELDFDPEL---AVERAVDTT
HcNarG   419 DRDGQNDPE-----------------------------------------SSI-------ELDFDPRL---SVERSVDLA
SsNarG   390 ARDGQHDAT-----------------------------------------ASI-------DLDFDPQL---AVERTVSTS
AoPcrA   351 DQPEKKGSP------------------------VGFLGRN----TFAFPKGYI------DLGDLDPAL---EGKFNMQLL
EcNarG   393 FRWGEK--------GKWNLEQRDGKTGEETELQLSLLGSQDEIAEVGFPYFGGDGTEHFNKVELENVLLHKLPVKRLQLA
SeNarG   389 QRWEEG--------KKWNLKLETE-DGTPIDPMLSMVESDYHVETIQFPYFDSSG---------DGIFERPIATRTIQLA
BcNarG   394 SRWDNE--------KKWNLRLEDEETGEKIDPRLSLLGMEESIGTVQIPYFSDDG---------NKVLERTIPVKKVMTE
BfNarG   430 HRFSAD------GEGRWNLELGD------LEPTLSLLGHHEDVAEVLLPRFDTVGQGG------RGDVPRGVPVRTV---
BpNarG   393 FRWGEAAHNGGEKVGRWNLEMKDSGSGRAIDPRLSLVDAHDEIVDVGFPYFGGEH---------EAVLARRVPAKRVALA

HmNarG   435 SG----RVQVRTVWAELR------------------DEL-ANWDPEMVHEETTVGKETYQRIAREFAEA-----DKAKII
HmaNarG  403 EG----SVAVRSVWENLT------------------DEL-SQYTPDVVHEETGVGENTYQRVAREFAEA-----DAAKII
HlNarG   410 DS---GQVSVRTVWDNLR------------------EEL-ATYTPEHVNEVTGVGRETHQEVAREFAEV-----DRAKII
HsNarG   411 DG----SVQVRSVWSHVT------------------EEL-SNYTPERVHELTRVGEETHQEIAREFANV-----DKGKII
HpNarG   431 DG----SVEVRTVWNELR------------------DEL-SRYDPETVHEMTGVGRETYQRVAREFAEV-----DRAKII
HaNarG   405 -D---GDVRVRTVWLRLR------------------DEL-ATYTPEYVNEESGVGQETHQRVAREFAQA-----ERAKII
HmuNarG  404 DG----EVAVRSVWENLR------------------EEL-SQYTPAFVHEETGVGENTYQRVAREFADA-----DAAKII
HuNarG   402 DG----EVAVTSVWNNLR------------------EEL-ANYTPEYVADETGVGKETHQKIAREFADV-----DRGKII
HrNarG   405 -D---GEVQVRSVWNNLN------------------DEL-ANYTPEYIYEETGVGEQTHQEIAREFAEA-----EKAKII
HliNarG  408 -D---GAVETRSVWLNLR------------------DEL-SAYTPERVNEITGVGEQTHQTIAREFAEV-----DRAKII
HcNarG   448 DGADADSVAVRSVWENLR------------------DEL-SEYTPEFVTDETTVGEETYQRVAREFAEA-----DKAKII
SsNarG   419 -D---GPVAVRSVWNELR------------------DEL-STYTPEHVHELTGVGRQTHQEIAREFADV-----DRAKII
AoPcrA   394 DG---KTVEVRPVFEILK------------------SRLMADNTPEKAAKITGVTAKAITELAREFATA-----KPSMII
EcNarG   465 DG---STALVTTVYDLTLANYGLER-GLNDVNCATSYDDVKAYTPAWAEQITGVSRSQIIRIAREFADNADKTHGRSMII
SeNarG   451 NG---EEVKIATVYDLMTSQYGVQR-FEHELEATSYDDASSKYTPAWQEQITGIKKELVTKVAKEFAQNAIDTGGRSMII
BcNarG   457 EG----EVFVTTVYDLTLANYGVNR-GLGGQEQKDFND-DVPFTPAWQEKITGVKRELIIQIAREFAQNAVDTNGRSMII
BfNarG   489 -----AGRLVTTVFDLLLAEYGVGREGLPGQWASGLDDADALYTPAWQEGITGVPGQAAARIAREFAQNAVDSGGRSMII
BpNarG   464 DG---TSALVATVYDLQMANYGVDQ-GLGGPNVAASYDDDIPYTPAWQEKHTGVARHLVIQVAREFADNADRTRGKSMVI

HmNarG   487 QGKGVNDWYHNDLGNRALQLLVTLTGNLGEQGTGLDHYVGQEKIWTFHGWKTLSFPTGKV---RGVPTTLWTYYHAGIL-
HmaNarG  455 HGKGVNDWYHNDLGNRAIQLLVTLTGNLGDPGTGLDHYVGQEKIWSFHGWKVLSFPTGNV---RGVPTTLWTYFHAGIL-
HlNarG   463 HGKGVNDWYHNDLGNRAIQLLVTLTGNLGRQGTGLDHYVGQEKIWTYSGWQSLSFPTGSV---RGVPTTLWTYYHSDII-
HsNarG   463 HGKGVNDWYHNDLGNRAIQLLVTLTGHIGRQGTGFDHYVGQEKIWTYNGWQQLSFPTESV---RAVPTTLWTYFHGDIL-
HpNarG   483 QGKGVNDWYHNDLGNRAIQLLVTLTGNLGRQGTGLDHYVGQEKIWTHHGWQTLSFPTGKV---RGVPTTLWTYYHAGIL-
HaNarG   457 HGKGVNDWYHNDLANRAIQLLVTLTGNLGRQGTGLDHYVGQEKMWASHGFSELSYPTGNV---RGVATTLWTYYHAGIL-
HmuNarG  456 HGKGVNDWYHNDLGNRAIQLLVTLTGNLGEPGTGLDHYVGQEKIWTFHGWKTLSFPTGSV---RGVPTTLWTYFHAGIL-
HuNarG   454 HGKGVNDWYHNDLGNRAIQLLVTLTGHIGRNGTGVDHYVGQEKIWTFNGWKTLSFPTGSV---RGVPTTLWTYYHAGIL-
HrNarG   457 HGKGVNDWYHNDLGNRAIQLLVTLTGNLGEQGTGLDHYVGQEKIWTFKGWKNLSFPTGSV---RGVPTTLWTYFHAGIL-
HliNarG  460 HGKGVNDWYHNDLGNRAIQLLVTLTGNLGRQGTGLDHYVGQEKIWTYNGWQKLSFPTGSV---RGVPTTLWTYYHAGIL-
HcNarG   504 HGKGVNDWYHNDLGNRAIQLLVTLTGNLGEQGTGLDHYVGQEKIWTFHGWQTLSFPTGSV---RGVPTTLWTYFHAGIL-
SsNarG   471 HGKGVNDWYHNDLGNRAIQLLVTLTGNLGRQGTGLDHYVGQEKIWTFHGWQTLSFPTGSV---RGVPTTLWTYYHCDVL-
AoPcrA   448 CGGGTQHWYYSDVLLRAMHLLTALTGTEGTNGGGMNHYIGQWKPAFVAGLVALAFPEGVN-KQRFCQTTIWTYIHAEVN-
EcNarG   541 VGAGLNHWYHLDMNYRGLINMLIFCGCVGQSGGGWAHYVGQEKLRPQTGWQPLAFALDWQRPARHMNSTSYFYNHSSQWR
SeNarG   527 MGAGINHWFNSDTIYRSILNLVLLCGCQGVNGGGWAHYVGQEKCRPIEGWNTIAFAKDWQGPPRLQNGTSWFYFATDQWK
BcNarG   531 VGAGINHWFNSDTIYRAVLNLVLLVGAQGVNGGGWAHYVGQEKFGPAKGWQTIAMAKDWQGPPKLQNGTSFFYFVTDQWR
BfNarG   564 MGAGTNHWFHSDTIYRSFLTLTNLCGTQGVNGGGWAHYVGQEKVRPITGWAHLANALDWSRPPRQMCQTTYWYMHADQWR
BpNarG   540 VGAALNHWYHNDMIYRGIINLLMMCGCIGQSGGGWAHYVGQEKLRPQFGWAPLAFALDWSRPPRQMNGTSFFYNHTSQWR

HmNarG   563 --------------DN---TDPDTAAKIRESIDKGWMPVYPEER------------------------------------
HmaNarG  531 --------------DN---TDPDTAEKIRESIDKGWMPVYPEER------------------------------------
HlNarG   539 --------------DN---VDEETADRIREAIDRDWMPVYPEER------------------------------------
HsNarG   539 --------------GN---VEPDTRQRIQESIDNGWMPVYPKER------------------------------------
```

```
HpNarG   559 --------------DN---TDPDTAAKIRESIDRGWMPVYPAER------------------------------------
HaNarG   533 --------------DN---TDEDTARRIRESVDNGWMPLYPAER------------------------------------
HmuNarG  532 --------------DN---TDPDTAEKIRESIDEGWMPVYPEER------------------------------------
HuNarG   530 --------------EN---TDAETRRKIEEAVEKDWMPVYPEER------------------------------------
HrNarG   533 --------------DN---TDPDTAAKIRESIEKGWMPLYPSER------------------------------------
HliNarG  536 --------------EN---TDPDTAAKIRESINKGWMPLYPSER------------------------------------
HcNarG   580 --------------DN---TDEDTAAKIRESIKKDWMPVYPSER------------------------------------
SsNarG   547 --------------DN---ADPETTRRVREAIDNDWMPVYPEER------------------------------------
AoPcrA   526 --------------DEIISSDIDTEKYLRDSITTGQMPNMPEQG------------------------------------
EcNarG   621 YETVTAEELLSPMADKSRYTGH-LIDFNVRAERMGWLPSAPQLGTNPLTIAGEAEKAGMN----PVDYTVKSLKEGSIRF
SeNarG   607 YEESNVDKLKSPLAENIKHQHP--ADYNVTAARMGWLPSYPQFNKNSLLFGEEAKDEGDDSNEAILQKAIESVKNKDTQF
BcNarG   611 YEDTPVGHLASPVEGNSRYQHH--GDYNVLTARLGWLPSYPTFERNGIELYKEAVAAGATTQEEIGKYVAQKLKEKELKF
BfNarG   644 YDRFGADTLAATT-GAGSFADMTTADAVALSQRLGWQPFFPQFDISSLDVADRAAEAGRE----TVPWLVDALKDGSVRF
BpNarG   620 HEKLAVGEILGPTADASKYAGMTLLDLNAKSERMGWLPSAPQLGRNPLDVVDEAERAGKE----PVAYAVEMLKSDKLAF

HmNarG   590 ----DNGSRPDPTTMFVWRGNYFNQA-KGNVA------------------------VEE------QLWPKLDLVVDINFR
HmaNarG  558 ----DDGNRPDPSTMFVWRGNYFNQA-KGNVA------------------------LEE------QLWPKLDLVVDINFR
HlNarG   566 ----DDGSRPDPSVLFMWRGNYFNQS-KGNVA------------------------IEE------RLWPKLDLIVDINFR
HsNarG   566 ----EDGSRPDPSVFFCWRGNYFNQS-KGGIA------------------------IEN------VLWPKLDLIVDINFR
HpNarG   586 ----EDGSRPDPSTMFVWRGNYFNQA-KGNVA------------------------VEE------ELWPKLDLVVDINFR
HaNarG   560 ----PNGSRPDPSVMLVWRGNYFNQA-KGNVA------------------------VEE------TLWPKLDLVVDINFR
HmuNarG  559 ----EDGSWPDPSTMFVWRGNYFNQA-KGNVA------------------------VEE------QLWPKLDLVVDINFR
HuNarG   557 ----GDGTRPDPSTMFVWRGNFFNQA-KGNVA------------------------VEE------VLWDKLDLVVDINFR
HrNarG   560 ----EDGTRPDPSILFCWRGNYFNQA-KGNVA------------------------VEE------ELWPKLDLVVDINFR
HliNarG  563 ----DDGGRPDPRVMFVWRGNYFNQA-KGNIA------------------------VEE------ELWPKLDLVVDINFR
HcNarG   607 ----EDGSRPDPSTMFVWRGNYFNQA-KGNVA------------------------VEE------ELWPKLDLVVDINFR
SsNarG   574 ----ADGSRPDPSILFVWRGNYFNQA-KGNVA------------------------VEE------QLWPKLDLVVDINFR
AoPcrA   556 ---------RDPKVFFVYRGNWLNQA-KGQKY------------------------VLE------NLWPKLELIVDINIR
EcNarG   696 AAEQPENGKNHPRNLFIWRSNLLGSSGKGHEFMLKYLLGTEHGIQGKDLGQQGGVKPEEVDWQDNGLEGKLDLVVTLDFR
SeNarG   685 AIEDPDLRKNHPKTLFVWRSNLISSSAKGQEYFMKHLLGARSGLMAEPN---EDDKPEEIKWRED-TEGKLDLLVSLDFR
BcNarG   689 AIEDPDNKNNFPRNLFVWRANLISSSGKGHEYFLKHLLGTTNGLMNDDS---DSLRPEEIKWHEEAPEGKLDLLINLDFR
BfNarG   719 AAEDIDAPENFPRIWSIWRANTLGSSAKGDQYFFRHLLGVDSSAAEEET--PEEFRPRDVRWREDAPIGKVDLMLTLDFR
BpNarG   696 ACDDPDNPANFPRNMFVWRSNILGSSGKGHEYFLKYLLGTQNALFSDEA---DALKPSEVQVR-DAAEGKLDLLAVLDFR

HmNarG   635 MDSTAMYSDIVLPTASHYEKHDLSMTDMHTYVHPFTPAVEPLGESKTDWQIFRELAQKIQEVATERGVEPISDRKFD---
HmaNarG  603 MDSTAMYSDIVLPAASHYEKYDLSETDMHTYVHPFTPAVEPLGEAKTDWEIFRLLAEKIQERAQERGVEPIEDRKFD---
HlNarG   611 MDSSALYSDIVLPTASHYEKHDLSMTDMHSYVHPFTPAVEPLGESKTDWQIFRELAAKIQEVATERGIDPIEDRSFD---
HsNarG   611 MDSTALYSDIVLPTASHYEKHDLNMTDMHSYVHPFTPAIEPLGESKTDWEIFRLIAKKVQEIATERNLSPIQDREFD---
HpNarG   631 MDSTAMYSDIVLPTASHYEKHDLSMTDMHSYVHPFTPAVEPLGESKTDWQIFRELAAKIQEVATDRDVEPIPDRQFD---
HaNarG   605 MDSTALYSDIVLPTASHYEKYDLSMTDMHSYVHPFTPAVEPLGESKTDWEIFRLLAEKIQEIATERGLAPVQDRKFD---
HmuNarG  604 MDSTAMYSDIVLPAASHYEKHDLNMTDMHTYVHPFTPAVEPLGEAKSDWEIFRLLAEKIQERAQERGVEPVEDRSFD---
HuNarG   602 LDSTALYADIVLPAASHYEKHDLNMTDMHTYVHPFTPAVEPLGESKSDWQIFRELAAKIQEIARDRDIDPIDDRKFD---
HrNarG   605 MDSTAMNADIVLPAASHYEKHDLSMTDMHTYVHPFTPAVEPLGDSKTDWQIFRELAAKIQALATERGIDPVPDRKFD---
HliNarG  608 MDSTALNSDIVLPTASHYEKHDLSETDMHSYVHPFTPAVEPLGESKTDWQIFRELAAKIQELAEERGTEPVDDRKFD---
HcNarG   652 MDSTALYSDIVLPAASHYEKYDLSMTDMHSYVHPFTPAVEPLGEAKTDWQIFRDLAEKIQEVAQERGVEPIDDRQFD---
SsNarG   619 MDSSALYSDIVLPSASHYEKHDLSMTDMHTYVHPFTPAVEPLGESKTDWQIFRELAAKIQELAEERGIEPVPDRKFD---
AoPcrA   596 MDSTALYSDVVLPSAHWYEKLDLNVTSEHSYINMTEPAIKPMWESKTDWQIFLALAKRVEMAAKRKKYEKFNDEKFK---
EcNarG   776 LSSTCLYSDIILPTATWYEKDDMNTSDMHPFIHPLSAAVDPAWEAKSDWEIYKAIAKKFSEVCVGH-LGKETDIVTLPIQ
SeNarG   761 MTATPLYSDIVLPAATWYEKHDLSSTDMHPFIHPFNPAIDPLWESRSDWDIYKTLSKAVSEMAKDYLPGKFKDVVTTPLG
BcNarG   766 MAGTALYSDIVLPASTWYEKHDLSSTDMHPFVHPFNPAIGSPWEARSDWNIFTSLSKAVSDLAKKIDLEPMKEVVATPLL
BfNarG   797 MTSHTLHSDVVLPASTWYEKHDLSTTDMHPFIHSFNPAISNPWESRTDWETWAAISERFSELARDH-LGTRTDVVALALQ
BpNarG   772 MSTTCLYGDIVLPTATWYEKDDLNTSDMHPFIHPLSEAVQPLWESKTDWEIYKTIAKKFSELAGPY-LGTRRDLVCTPLL

HmNarG   712 --REIDLQSVYDDYVRDWETGE----------------------------------------------------------
HmaNarG  680 --RTIDLTTIYDDYVRDWETGE----------------------------------------------------------
HlNarG   688 --REIDLQSVHDDYVRDWETGE----------------------------------------------------------
HsNarG   688 --RQIDLTSVHDDYVRNWPDDE----------------------------------------------------------
HpNarG   708 --REIDLRSIHDDYVRDWASDE----------------------------------------------------------
HaNarG   682 --REIDLQSVYDDYVRDWLTGE----------------------------------------------------------
HmuNarG  681 --REIDLTTIYDDYVRDWETGE----------------------------------------------------------
HuNarG   679 --RQIDLQSVHDDYVRDWVSDE----------------------------------------------------------
HrNarG   682 --REIDLQSVHDDYVRDWQTGE----------------------------------------------------------
HliNarG  685 --RRIDLRSVRDDYVRDWLDDE----------------------------------------------------------
HcNarG   729 --REIDLQSIYDDYVRDWESDE----------------------------------------------------------
SsNarG   696 --RDIDLQSVHDDYVRDWETGE----------------------------------------------------------
AoPcrA   673 --WVRDLSNLWNQM------TM----------------------------------------------------------
EcNarG   855 HDSAAELA-QPLD-VKDWKKGECDLIPGKTAPHIMVVERDYPATYERFTSIGPLMEKIGNGGKGIAWNTQSEMDLLRKLN
SeNarG   841 HDSKQEIS-TEYGIVKDWSKGEIEGVPGKTMPNFSIVERDYTQIYDKFVTVGPKLEKGKIGAHGVSYSVSEEYEELKSIV
BcNarG   846 HDTPQELA-QPLGKIKDWSKGECEPIPGKTMPQIHVVERDYKTIYDKMTALGPNAGKQPIGTKGISWSAEKEYEQLKSKL
```

```
BfNarG   876 HDTPDAMA-TPHGRVRDWKKGECEAVPGLTMPALVEVERDYTQVHAKFTSIGPLLEEKGMTTKGLTYDVSQYVAELGALN
BpNarG   851 HDTPGELG-QPFE-PKDWRRGECDLIPGKTAPSMTVVERNYADIYKKFTSIGPLLDKLGNGGKGINWNTEHEVKEIGALS

HmNarG   732 --------------EGALAEDRAACEYILEHSEESNPA-----------------------DSDEQITFADTVEQPQRLL
HmaNarG  700 --------------EQGLVEDKAAAEFILEHSEETNPE-----------------------GSDEQITFDDIDEQPQRFL
HlNarG   708 --------------AGALAEDRAACEYILENSEETNPS-----------------------DSDERITFEDIDEQPRRFP
HsNarG   708 --------------SGALKEDKAASEFILDHSEETNPE-----------------------GSDSQITFDDTVEHPRRFE
HpNarG   728 --------------RGALAEDRAACEYILDHSEESNPE-----------------------GTDERITFADTVEQPRRLL
HaNarG   702 --------------EGALEADRAACEFILDHSEETNPQ-----------------------DVDDRITFDQIEEQPRRFL
HmuNarG  701 --------------EGALEDGREASEFVLEHSEESNPA-----------------------DSDEQITFADTVEQPQRLE
HuNarG   699 --------------DGALEEDRAACEAILEHSTETNPD-----------------------DG-GEITFADTVDQPQRFE
HrNarG   702 --------------DGALAQDKAAAEYILEHSEESNPS-----------------------DTDEQLTFDEIDEEPKRLL
HliNarG  705 --------------AGALAEDEAAAEFILENSEETNPE-----------------------GTDERITFADVDEQPRRLL
HcNarG   749 --------------EGALAEDRAACEYILEHSEESNPE-----------------------GTDEQITLDDTIDQPQRLL
SsNarG   716 --------------PGGLADDRAAAEYILDHSEETNPE-----------------------GTNERITFDDIDDQPRRFL
AoPcrA   687 --------------DGKLAEDEAAAQYILDNAPQS-----------------------------KGITIQMLREKPQRFK
EcNarG   933 YTK----AEGPAKGQPMLNTAIDAAEMILTLAPETNGQVAVKAWAALSEFTGRDHTHLALNKEDEKIRFRDIQAQPRKII
SeNarG   920 GTWNDDNTISVKNDRPRIDTARKVADVILNISSATNGKLSQKSYEDLENQTGMELKDISKERASEKISFLNITSQPREVI
BcNarG   925 GVVRTDT---IAKGCPDIKEAINAAEAVLTLSSTTNGHMAVKAWEALEKQTDLKLRDLAEEREEECFTFEQITAQPKTVI
BfNarG   955 GVH----RSGPAAGRPKIETDLQACEFILALSGTTNGHMATQGFRTLEKRTGTTMHDLAAEHEGKRISFADTKAAPVPVV
BpNarG   929 DTVA---EPGVSRGRPRLDTAIDAAEMILTFAPETNGHVAVKAWDALSKITGRDHTHLAVGREHDKIRFRDVQAQPRKII

HmNarG   775 EAGDHWTSDIEDGEAYAPWKDFVQDKNPWPTVTGRQQYYIDHDWFLELGEELPTHKEGPE-----------N-TGGDYPM
HmaNarG  743 EAGDHWSSDIKDDEAYVPWQDYVHDKNPWPTFTGRQQYYIDHDWYLELGEELPTHKEGPE-----------N-TGGDYPL
HlNarG   751 KAGDHWTSDIEDGEAYTPWQSYVQDKEPWPTFTGRQQYYIDHDWFLDLGEELPTHKDAPT-----------LQDKSEYPL
HsNarG   751 AAGDHWTSPLEEGKPYTPWKRYVQQKNPWPTFTGRQQYYIDHDWFLDLGEAVPTYKEPEV-----------LQSEQEYPL
HpNarG   771 EAGDHWTSDIEEGEAYMPWKDFVQEKNPWPTVTGRQQYYVDHDWFLELGEQLPTHKEGPT-----------K-TGGDYPM
HaNarG   745 ATGDHWTSPIEEGEAYTPWKQFVQDKEPWPTFTGRQQYYIDHDWFLDLGEELPTHKQPTN-----------EQDPDEYPL
HmuNarG  744 AAGDHWTSDIEDGAPYVPWQDYVQDKQPWPTFTGRQQYYVDHDWFLELGEELPTHKEGPQ-----------D-TGGDYPL
HuNarG   741 AAGDHWTSDIEDGTAYAPWKDFVQDKEPWPTLTGRQQYYIDHDWFLDVDEQLPTHKRPVE-----------TNDQSEYPL
HrNarG   745 ATGDHWTSDIEDGEAYTPWKRYVQDKEPWPTFTGRQQYYIDHDWFLELDEQLPTHKQGPV-----------LQEKSEYPL
HliNarG  748 EAGDHWSSDIEEGEAYTPWKDYVQDKNPWPTFTGRQQYYIDHDWFLELDEQLPTHKEAPV-----------LQEKSEYPL
HcNarG   792 EAGSHWTSDIEDGEAYTPWQNYVQDKEPWPTFTGRQQYYVDHDWYLELGEELPTHKEGPA-----------N-TGGSYPL
SsNarG   759 ATGDHWTSDIEEGEAYAPWKDYVQDKQPWPTFTGRQQYYIDHDWFLELDEQLPTHKQAPT-----------LQDKADYPL
AoPcrA   724 ---SNWTSPLKEGVPYTPFQYFVVDKKPWPTLTGRQQFYLDHDTFFDMGVELPTYKAPI--------------DADKYPF
EcNarG  1009 SS-PTWSGLEDEHVSYNAGYTNVHELIPWRTLSGRQQLYQDHQWMRDFGESLLVYRPPIDTRSVKEVIGQKSNGNQEKAL
SeNarG  1000 PT-AVFPGSNKDGRRYSPFTTNVERLVPFRTLTGRQSYYIDHEVFQQFGESLPVYKPTLPPMVFGARDKKVKGGQDTLVL
BcNarG  1002 TS-PAFTGSEKGGRRYSPFTTNVERLIPWRTITGRQSFYLDHDMMKEFGETMATFKPILQHKPFRKSRPEVEG--KEITL
BfNarG  1031 TS-PEWSGSETGGRRYSPFTINVERKKPWHTLTGRQHFYLDHDWMLEMGEALPVFRPPLDMTALFGEKTPGHTDGTSISV
BpNarG  1006 SA-PTWSGLESEEVSYNAGYTNVHELIPWRTLTGRQQFYQDHRWMLDFGEGSCAYRPAIDTKTVAPLHKRMPNGQPELVL

HmNarG   843 EYNTPHGRWAIHSTWRDSEKLLRLQRGEPLLYLHPEDAEERGIEDGDSVEVFNDLAEVELQAKIYPSSQRGTARMYFAWE
HmaNarG  811 SYNTPHGRWSIHSTWRDDTKMLRLQRGEPVVYLNPEDAQQRGIEDGDTVEVYNDLGSVEVQAKIYPSSEPGTVRHFFSWE
HlNarG   820 RYNTPHGRWSIHSTWRDSEKMLRLQRGEPIVYLNPEDMDERGIEDGDTVRIYNDVGEVEIQAKRYPSGEPGTARMYFAWE
HsNarG   820 QYNTPHGRWSIHSTWRDNKHMLRLQRGEPIVYMHPDDAEERGIEDGDTVRIYNDLDEIEVSVKIYPSAQPGVAKLYFAWE
HpNarG   839 EYNTPHGRWSIHSTWRDNEKLLRLQRGEPVVYLHPEDAAERGIEDGDAVEVFNDLAEVELQAKLYPSSQRGTARMYFAWE
HaNarG   814 RYNTPHGRWSIHSTWRDNETLLRLQRGEPVVYLNPDDAAARDVEDGDTVRLYNDLGSVELQVKIYPSGEPGTARMFFAWE
HmuNarG  812 SYNTPHSRWSIHSTWRENTKMLRLQRGEPTVFLNPEDAEERGIEDGDTVEVYNDMGSVEVQAKIYPSGDPGTVRHFFSWE
HuNarG   810 RYNTPHGRWSIHSTWRDSEKMLQLNRGEPVVFIHPEDAKHRGIEDGDTVEIYNDLATIEANAKLYPASEPGTVRHYFAWE
HrNarG   814 GYNTPHGRWSIHSTWRDNTKMLRLQRGEPVVYLNPEDAAERGIEDGDTVRVYNDLGSVEVQAKIYPSSEPGTVRHFFSWE
HliNarG  817 GYNTPHSRWSIHSTWRDSTKMLRLQRGEPTVYLNPEDAEVRDIEDGDTVRVYNDLDSVEVQAKIYPSGEPGTVRHFFSWE
HcNarG   860 SYSTPHSRWSIHSTWRDNETMLNLQRGEPTVFLNPQDAEERGIEDGDTVEVFNDLGSVEVQAKIYPSSEPGTVRHFFSWE
SsNarG   828 RYNTPHGRWSIHSTWRDSERMLRLQRGEPVVYLNPDDMAERGIEDGDTVRVYNDLGEVEVQAKRYPSSEPGTARMYFAWE
AoPcrA   787 RFNSPHSRHSVHSTFKDNVLMLRLQRGGPSIEMSPLDAKPLGIKDNDWVEAWNNHGKVICRVKIRNGEQRGRVSMWHCPE
EcNarG  1088 NFLTPHQKWGIHSTYSDNLLMLTLGRGGPVVWLSEADAKDLGIADNDWIEVFNSNGALTARAVVSQRVPAGMTMMYHAQE
SeNarG  1079 RYLTPHGKWNIHSTYQDNERMLTLFRGGPVVWISNEDAADHGINDNDWLEVYNRNGVVTARAVTSHRMPRGTMFMYHAQD
BcNarG  1079 NYLTPHNKWSIHSMYFDSLPMLTLFRGGPTVWMNKEDAAEAGVADNDWIECFNRNGVVVARAVVTHRIPRGMAFMHHAQD
BfNarG  1110 RYLTPHNKWAIHSMYQENFFMMNLSRGGQNIWMSVEDAEAVGITDNDWVEAVNRNGVVSARAVVSHRMPRGTAFMHHGQE
BpNarG  1085 NWITPHQKWGIHSTYSDNLRMLTLSRGGPHVWISEAEAQQAGIRDNDWVEVFNVNGTLTARAVVSQRVPAGMCLMYHAQE

HmNarG   923 RFQFD--------SDSNFNSLVPMYMKPTQLVQYPEDSGEHLHFFPNYWGPTGVNSDVRVDVRKAGGGDE----------
HmaNarG  891 KFQYP--------DRDNFNTLVPMYMKPTQLVQYPEDTGEHLHFFPNYWGPTGVNSDVRVDVRPSGGDTE----------
HlNarG   900 RFQFP--------DRNNFNSLVSMYMKPTQLVQYPEDTGEHLHFFPNYWGPTGVNSDVRVEVEKVADADQEGS-------
HsNarG   900 RFQFP--------SRGNFNTLVGMYMKPTQLVQYPADSGEHLEFVPNYWGPTGVNSDVHVEVELVEDEATGEGTATTTAQ
HpNarG   919 RFQFP--------TDSHFNSLVPMYMKPTQLVQYPEDTGEHLYFFPNYWGPTGVNSDVRVDVRKKGGDGG----------
HaNarG   894 KFQFA--------SRNNFNTLVPMYMKPTQLVQYPEESGEHLSFSPNYWGPTGVNSDVRVEVEKIDGGDGE---------
HmuNarG  892 KFQYP--------GRDNFNTLVPMYMKPTQLVQYPEDTGEHLYFFPNYWGPTGVNSDVNVDVRLTDGQSGSGDEQ-----
HuNarG   890 RYQYP--------SRNNFNSLIPMYMKPTQLVQYPEDSGEHLHFFPNFWGPTGVNSDVRCDIRPKEGGDD----------
```

```
HrNarG    894 KFQYP--------DRDNFNTLVPMYMKPTQLVQYPEDTGEHLHFFPNYWGPTGVNSDVRVEVERVDDGSGSTGGQGGD--
HliNarG   897 RFQYP--------DRNNFNSLVPMYMKPTQLVQYPEDTGEHLHFFPNYWGPTGVNSDVRIEVEKVEEGAASGDDARSV--
HcNarG    940 KFQYP--------DRDNFNTLVPMYMKPTQLVQYPEDTGEHLYFFPNYWGPTGVNSDVRVDVRPAEQGESEGESDGDG--
SsNarG    908 RFQYP--------DRGNFNSLVPLYMKPTQLVQYPEDTGEHLHFFPNYWGPTGVNSDVRVEVERVGGGDSDDDGDDGD--
AoPcrA    867 LYMDL--------LTGGSQSVCPVRINPTNLVG----NYGHLFFRPNYYGPAGSQRDVRVNVKRYIGATPISF-------
EcNarG   1168 RIVNLPGSEITQQRGGIHNSVTRITPKPTHMIG----GYAHLAYGFNYYGTVGSNRDEFVVVRKMKN-IDWLDGEGNDQV
SeNarG   1159 KHIETPGSEITDTRGGSHNAPTRIHLKPTQLVG----GYAQISYHFNYYGPIGNQRDEYVAVRKMKE-VNWLED------
BcNarG   1159 RHINVPGTKLTSNRGGTHNSPTRIHVKPTHMIG----GYGQLSYGFNYYGPTGNQRDLNVVIRKLKE-VDWLED------
BfNarG   1190 RTVNVPLTERDGKRGGITNSLTRIMIKPSHLIG----GYAQLSFAFNYYGPTGNQRDEVTMIRKRTAPVEY---------
BpNarG   1165 KIVNVPGAQTSGKRGGIHNSVTRTVTKPTHMIG----GYAQQAYGFNYYGTVGSNRDEYVIVRKMNR-VDWLEEPLNEGA
```

```
HmNarG        --------------------------------------------------------------------------------
HmaNarG       --------------------------------------------------------------------------------
HlNarG        --------------------------------------------------------------------------------
HsNarG    972 DQNGSSGTTA-----------NQTTANSGTMNTTSGTTDGTASNETTTSSESTSTNATASGNTSDLDVIPGDSPDEASGG
HpNarG        --------------------------------------------------------------------------------
HaNarG        --------------------------------------------------------------------------------
HmuNarG   959 --RESVGVRPAGGDDQ----------------------------------------------------------------
HuNarG        --------------------------------------------------------------------------------
HrNarG    964 -AQ-----------------------------------------------------------------------------
HliNarG   967 -GDGLSLERGDGTGPGDPAAGDAETDSRGALGRVSDLLGGDD--------------------------------------
HcNarG   1010 -EETAADLRLADGGDPPDGS--TVDAA----------DGGEQS-------------------------------------
SsNarG    978 -DGGSGG---DGGGDGGEGA------------------------------------------------------------
AoPcrA        --------------------------------------------------------------------------------
EcNarG   1243 QE--SVK-------------------------------------------------------------------------
SeNarG        --------------------------------------------------------------------------------
BcNarG        --------------------------------------------------------------------------------
BfNarG        --------------------------------------------------------------------------------
BpNarG   1240 EQ------------------------------------------------------------------------------
```

Figure S1. Complete alignment of NarG sequences *Escherichia coli* (*Ec*NarG; Protein NCBI ID: WP_000032939.1; UniProt: P09152), *Bacillus cereus* (*Bc*NarG; Protein NCBI ID: WP_000729051.1), *Staphylococcus epidermidis* (*Se*NarG; Protein NCBI ID: WP_011082781.1), *Brachybacterium faecium* (*Bf*NarG; Protein NCBI ID: WP_015775897.1), Burkholderia pseudomallei (BpNarG; Protein NCBI ID: WP_004555870.1), *Azospira oryzae* (*Ao*PcrA; Protein NCBI ID: WP_014235273.1; UniProt: G8QM55), *Halococcus salifodinae* (*Hs*NarG; Protein NCBI ID: EMA48671.1), *Halorhabdus utahensis* (*Hu*NarG; Protein NCBI ID: WP_012795090.1), *Haloferax mediterranei* (*Hm*NarG; Protein NCBI ID: WP_004056332.1), *Halogeometricum pallidum* (*Hp*NarG; Protein NCBI ID: WP_008385261.1), *Halosimplex carlsbadense* (*Hc*NarG; WP_006885135.1), *Haloarcula marismortui* (*Hma*NarG; Protein NCBI ID: WP_011223493.1), *Halomicrobium mukohataei* (*Hmu*NarG; Protein NCBI ID: WP_015763420.1), *Halogranum amylolyticum* (*Ha*NarG; Protein NCBI ID: WP_089826390.1), *Halorientalis regularis* (*Hr*NarG; Protein NCBI ID: WP_092694237.1), *Halorubrum lipolyticum* (*Hli*NarG; Protein NCBI ID: WP_008003395.1), *Halobellus limi* (*Hl*NarG; Protein NCBI ID: SEF60617.1), and *Salinigranum salium* (*Ss*NarG; Protein NCBI ID: WP_152042447.1). The TAT consensus sequence is underlined, twin arginine 'RR' is highlighted in yellow, hypothetical amino terminal residue of *Haloferax mediterranei* NarG is shown in green. His and Cys residues involved in FS0 cluster coordination are highlighted in cyan. The conserved residues in the gate substrate tunnel are highlighted in red, the catalytic aspartate is highlighted in gray whereas Lys that could form an electron transfer pathway bridge between FS0 and MoCo cofactor are in magenta. The amino acids with a possible role in substrate binding, reduction potential regulation of FS0 and proton donor during catalysis are highlighted in olive. The percentages of identity among haloarchaeal NarGs are comprised between 70% and 86%, with a coverage between 96% and 100%. The percentages of identity between haloarchaeal and bacterial NarGs are ranged between 34% and 36%, with a coverage between 91% and 94%.

```
HmNirK      1 ------MLSTTRRRTL---QLLGLGG-VASLAGCASEAPTAAQSL-------DQT---EEPTPAQQESPKIVEQVAANPT
RpNirK      1 MSRLHTVRSLMRAGLI-----SVVLGALLA-----GTAAR------------AASAKLPGDFGPPRG--EPIHAVLTSPP
NgAniA      1 ----------MKRQAL-----AAMIASLFALAACGGEPAAQAPAETPAASAEAASSAAQATAETPAGELPVIDAVTTHAP
HliNirK     1 -----MSQDLSRRKMV---AALGIGA-VGAVAGCIGAPVNAEPTP-------AAETTPAVTPELDPAREVDADRIAADPT
NbNirK      1 ---MTHSATIDRRRML---QVLGATG-AAAIAGCIATENTAPDEG-------NGSSADEIDDGLPAAESVDVDSVLADPT
NaNirK      1 MTTVATQPTTNRRAVI---HTIGGAG-AIALAGCLSDDPPEADSV-------ESEADTAPEEERPAAEPVDLDRIAADPM
HhNirK      1 ----MTTIQANRRQFM---QAIGATG-AVAVAGCLGNDDVSGS-----------STTTESEEGLPAAEAVDVDRVARDPT
HasNirK     1 --MTRRTHSKPRRRFL---QTAGAAT-ALAVAGCLGSGSDGDSADRS----TGDDTDSTDEASATAATSVDVDRIARDPT
HaeNirK     1 ------MFRSTRRKVL---QTLGVTGAAGSLAGCPAAPQSDTDP-----------------TPQVDGMPKEVDRVASDPT
SsNirK      1 ------MVRTTRRSVL---EALGVGG-AAAVAGCAANAPTAADTE-------RQR---TAME---QNAKPTVDRVAADPT
HlNirK      1 ------MFATTRRRTL---QALGLGG-AASLAGCASNAPTAEGST-------A-T---DAPEQTTTPKPVDTDRIAADPT
HpNirK      1 -----MTYTTTRRRVL---QGMAVGT-AGALAGCTVGAPLDDVQV-------AEPLNVGFEPSLAAAKPVDVDRVAADPR
HaNirK      1 ------MQASTSAFVLVAFVGEGV-G-AVAVAGCLSTTNTGQISV--------DS--VATPGSLTGALPVDVDRIAADPT
HmaNirK     1 ---MSTIPTATRRRVL---EALGVGT-A-ALAGCASAPGAKEQAT--------EAETTPQEPAMNAAQQTDVDRIAADPT
HuNirK      1 ---MSSIPVATRRRVL---QALGVGG-AAALAGCGASSDTDDQ-P--------AENTSPTEPHMNDPQMTDVDRIAADPT

HmNirK     61 DIPDPITRSEPTEVDVTLRPEEVTAEVEEGVTFTYMTYNGQVPGPFIRVRQGDTVNLTFENPEEN-SMPHNVDFHAVAGP
RpNirK     57 LVPPPVNRTYPAKVIVELEVVEKEMQISEGVSYTFWTFGGTVPGSFIRVRQGDTVEFHLKNHPSS-KMPHNIDLHGVTGP
NgAniA     66 EVPPAIDRDYPAKVRVKMETVEKTMKMDDGVEYRYWTFDGDVPGRMIRVREGDTVEVEFSNNPSS-TVPHNVDFHAATGQ
HliNirK    65 DVPAPVDWDEPRDHDIALRTKELVAEIEPGVTFKYMTFEGQVPGPMIRVRRGDTVNLRFEVPADDNSDIHNVDFHAVYGP
NbNirK     67 DIPAPVDWDEPREHDITIECTEHIAEIEPGVTFHFMTFEGQVPGPMVRVREGDTVNLTFRVPEESNMYAHNIDFHAVYGP
NaNirK     70 DIPPPVDWDEPRTHEITLESIEVTAEVEPGVTVDFMTYDGQIPGPMVRVREGDTVELTFAVPEEHNVDVHNVDAHAIYGP
HhNirK     62 DIPAPVDWDEPREHDITLETEQMTAEIEPGVTFDFMTFGGQIPGPMVRVRRGDRVNLRFEVPEDINMDLHNVDFHAVYGP
HasNirK    71 DIPDPVDWTEPREHDITIETERLTAEIEPGVTFDYMTFEGQVPGPMIRVRQGDRVNLTFDVPEDLNVEAHNMDFHAVYGP
HaeNirK    55 DVPDPIDRDQPKTHDITLQAQEVIAPIEEGNTFHFMTFDGQVPGPMIRVRQGDTVNLTFENLKSS-NLPHNVDFHAVYGT
SsNirK     58 DIPGPIQRSTPTTVDVTLRPEEVTAEIEDGVTFDYMTYNGQVPGPFIRVRKGDTVDLTFENPSGN-ALPHNVDFHAVAGP
HlNirK     60 DIPDPIDRDSPAEVDVTLRTEEVVAEVEEGVTFTYMTYNGRVPGPFIRVRQGDTVNLTFENAESN-SLPHNVDFHAVAGP
HpNirK     65 DIPKPITRSKPATVPVELETREAVAEIEPDVTFQYMTFNGQVPGPFIRTRVGDTVEVTIRNHESS-AMAHNVDFHACRGP
HaNirK     63 AIPGPIMRTTPETVVVELETRELVAEVEPGVTFTYMTFDNQIPGPFIRTRVGDTVDLTVTNHPDN-SMPHNIDLHSVRGP
HmaNirK    65 AIPDPIDRSEPKTVSVEMTTKEQVAEIEPGVTYTYMTFGDQIPGPMIRVRRGDTVELTITNEEGN-SMPHNIDLHAVRGP
HuNirK     65 DVPDPIDRSSPATVNVELETRELVAEVEPGVTYTYMTFDNQVPGPLIRVRKGDTVNMTVTSHEDN-TMPHNIDLHAVRGP

HmNirK    140 GGGAEATMTNPGE-TVKIRFKATYPGAYIYHCAVPNMDMHISAGMFGLILVEPPEGLPEVDKEVYIGQHELYTDKKAGKK
RpNirK    136 GGGAASSFTAPGH-ESQFTFKALNEGIYVYHCATAPVGMHIANGMYGLILVEPPEGLPKVDHEYYVMQGDFYTAGKYREK
NgAniA    145 GGGAAATFTAPGR-TSTFSFKALQPGLYIYHCAVAPVGMHIANGMYGLILVEPKEGLPKVDKEFYIVQGDFYTKGKKGAQ
HliNirK   145 GGGAVDTTLVPGDEAAELSFRAEFPGLFTYHCAVPAMDHHVSSGMFGAILVEPEAGLPAVDRELYLGQHELYTKGDLGEK
NbNirK    147 GGGAEHTTLRPGDEEATISARMDYAGSHIYHCAVPNMDHHISAGMFGAILVEPKDGLPEVDRELYLGQHELYTDGDTGEE
NaNirK    150 GGGAEATTLSPGDEPARLTFRAEFPGVHIYHCAVPNMDQHISLGMFGAILVEPAEGLPEVDREFYVGQHELYTDGEAGEE
HhNirK    142 GGGADATTIAPGDDAAEISFTAEYAGAFIYHCAVPNMDHHISAGMFGTILVEPEEGLPEVDHEFYLGQHEIYTEGEAGEE
HasNirK   151 GGGADATTIAPGDDPAQISFTAEYAGVFIYHCAIPNMDQHISSGMFGSILVEPEDGLPEVDREFYLGQHEIYTDGDLGEE
HaeNirK   134 GGGSEATDANPGE-TNTVKFQARYPGAFIYHCAVPNLDYHISSGMFGMIVVEPEDGFPEVDREFYLGQHEIYTQQHHGAE
SsNirK    137 GGGAEATMTAPGE-TARLRFKATYPGAYIYHCAVPNLDMHISAGMFGIILVEPEEGMPAVDHELYLGQHEIYTDKEAGES
HlNirK    139 GGGAEATTTAPGE-SADLRFQATYPGAYIYHCAVANMDMHISAGMFGLILVEPPEGMPEVDHEIYIGQHELYTNESAGEE
HpNirK    144 GGGAEATTVNPGE-EKRLRFKVTYPGAFVYHCAVANVDYHISSGMFGLILVEPEEGLPAVDREFYLGQMEVYTNGAAGDE
HaNirK    142 GGGAEDTMVMPGE-TKRITFKVTYPGLFVYHCAVPNLDYHISAGMFGAILVEPEEGLPPVDHEFYLGQHELYTTGETGEQ
HmaNirK   144 GGGAEASMVTPGQ-TKTFRFKATYPGAFIYHCAVPNLDMHISSGMFGMILVEPKEGLPEVDHEFYFGQHELYTTGDTGEK
HuNirK    144 GGGAEASMVAPGE-TETFQFKATYPGAFIYHCAVPNLDYHIASGMYGLILVEPEDGLPEVDHELYFGQNELYTTGDVSQD

HmNirK    219 GKHNFDFEAMRNEEPTYVVMNGEKYAWTDAG-RGPAATVNTGETVRVFFVDGGPNLSSSFHPIGSVWETLYPDGSLSTDP
RpNirK    215 GLQPFDMEKAIDERPSYVLFNGAEGALTGDK----ALHAKVGETVRIFVGNGGPNLVSSFHVIGAIFDQVRYEGGTN--V
NgAniA    224 GLQPFDMDKAVAEQPEYVVFNGHVGAIAGDN----ALKAKAGETVRMYVGNGGPNLVSSFHVIGEIFDKVYVEGGKL--I
HliNirK   225 GHHAFDHGAMLDEDPTYVVFNGEHHGFTEDR-RGGI-GAAVGESVRVFFVNGGPNQSSSWHPIGNVWSRLYRDGDLVSPP
NbNirK    227 GHHSYDFDAGAAEDPTYVLFNGEAYGLTEDGTHGPM-HAEVGETVRLHFANGGPNLLSSVHPIGNVFSRLYRDGDLLSDP
NaNirK    230 GHHAYDFDATADEDPTYVLLNGEVGALTEDG-HGPM-YAEVGETVRVYFANGGPNLTSALHPIGNVWSRYYRDGDLLSEP
HhNirK    222 GHHGFDFDAMQSEDPTYVTFNGQAYAFTPDG-LGPM-KANTGETARVYFANGGPNLTSALHPIGNVWSRYYRDGDLISEP
HasNirK   231 GHHGFDFDAMLAEQPTYVVFNGQAYGFTEDG-VGPM-HAEVGETARVYFANGGPNLLSSWHPIGNVWSRFYRDGDLLTEP
HaeNirK   213 GRLNFDIEGMMNEEPTYVLFNGEKYPYIPDK-YGSL-EAETGETVRVFLVVGGPNYSSNFHPIGNIWKRAYRDGAVVDSP
SsNirK    216 GKHRFDMQAMKREEPTYVVMNGEQYALTPDG-HGTAATVSTGDTVRTYFVTGGPNLTSSFHPIGNVWEELYPEGSLTTRP
HlNirK    218 GHHEFDMASMRSEEPTYVLMNGEKYAWTPAG-RGPAVTAGTDETVRVFFVDGGPNLASSFHPIGSVWEELYPDGSLSTEP
HpNirK    223 GHHEFDFETMAAEDPTYVLINGEKYAIGPQG-YDEM-RVRTDETVRIYYAVGGPNQFSSFHAIGGVWDEVYPQGSLASEP
HaNirK    221 GHHEFDFEAMAREDATYVLINGEKYAIGPQG-YNDM-QMTVGETARVYFAVGGPNLLSSFHPIGSVWDEVYPQGAIGSDP
HmaNirK   223 GHHDFDMEAMAAEEPTYVLMNGEKYAITPDR-HGSP-SMQVGETARVYFVTGGPNLDSSFHPIGSVWDEVWQQGSIAGPP
HuNirK    223 GHHDFDMDAMTAEEPTYVLMNGESRAITENR-YGPV-TVDVGDTARVYFVNGGPNLTSSFHPIGCVWDEVHPQGGIGGPP
```

```
HmNirK   298 QTHIQTRLVPPGSTTVATMSSPVPGDFKLVDHSLSRVTRKGCMAVIRAEGPEDPEIFDPNPE------------------
RpNirK   289 QKNVQTTLIPAGGAAVVKFTARVPGSYVLVDHSIFRAFNKGAMAILKIDGAENKLVYSGKELDSVYLGDRAAPNMSAVTK
NgAniA   298 NENVQSTIVPAGGSAIVEFKVDIPGNYTLVDHSIFRAFNKGALGQLKVEGAENPEIMTQKLSDTAYAGSGAASAPAASAP
HliNirK  303 GRYVETTAVAPGTATVGEMELPIPGPIKIVDHALSRAGRRGALAVVDVEGEPNSDVYDPGSDE-----------------
NbNirK   306 ARNVETTPVAPGTVTMAEMEMKVPGPVKIVDHALSRAGRKGALAVIDVDGEANPDIYNPDA-------------------
NaNirK   308 GRNVETNPVAPGTVVAGEMELPVPGPVKLVDHALSRVVRKGMIGVIDVEGEPNPAVYDDES-------------------
HhNirK   300 DRNIETAPVAPGTTTVGEMEFPVPGPVKIVDHALSRVVHKGALAVIDVEGEENPEVYEEDPQ------------------
HasNirK  309 DRNIETAPVAPGTTAAAEMEFPVPGPVKIVDHALTRAGRRGALAVIDVEGEEQPEIYDPNP-------------------
HaeNirK  291 ERYVQTMKVPPGSCMIGTMDLPVPERIYLVDHALSRYARRGLGAYLDITGEERPDIYDPSPDMDASEDEEGPFY------
SsNirK   295 ETHVQTKPVAPGSTTIATMSFPVPGNFKLVDHALSRVARKGCMAIVTAEGEERPDIFDPNPEQ-----------------
HlNirK   297 QTHIQTREVPPGSTAIATMNSPVPGDFKLVDHSLSRVARKGCMAVVRAEGEERPEIFDPDPA------------------
HpNirK   301 HRYVQTTPVLPGSAAVVTARFPVPGDYKLVDHALSRVARKGALAVLRAEGPANPDLFDPEDGDE----------------
HaNirK   299 HRFVQTTPVLPGSAVIAILSAPVPGPIKLVDHALSRVARKGCLAAIDVQGEEDPEIYDPEPAQG----------------
HmaNirK  301 NRYVQTTPVKPGSCAIATLHAEVPGPIKLVDHALSRVARKGTMAIINREGAANPDVFEPEA-------------------
HuNirK   301 HRNIQTTPVMPGSATIATMHFEVPGPVKLVDHALSRVARKGLLAVVEAEGDARPDLFDPDPD------------------

HmNirK       --------------------------------------------------------------------------------
RpNirK   369 ATQASVSGTLTVQDQVQAGRALFAGTCSVCHQGNGAGLPGVFPPLAKSDFLAADPKRAMNIVLHGLNGKIKVNGQEYDSV
NgAniA   378 AASAPAAS--A------SEKSVY---------------------------------------------------------
HliNirK      --------------------------------------------------------------------------------
NbNirK       --------------------------------------------------------------------------------
NaNirK       --------------------------------------------------------------------------------
HhNirK       --------------------------------------------------------------------------------
HasNirK      --------------------------------------------------------------------------------
HaeNirK      --------------------------------------------------------------------------------
SsNirK       --------------------------------------------------------------------------------
HlNirK       --------------------------------------------------------------------------------
HpNirK       --------------------------------------------------------------------------------
HaNirK       --------------------------------------------------------------------------------
HmaNirK      --------------------------------------------------------------------------------
HuNirK       --------------------------------------------------------------------------------

HmNirK       ---------------------------------------------------
RpNirK   449 MPPMTQLNDDEVANILTYVLNSWDNPGGRVSAEDVKKVRAQPAPAKAVAEH
NgAniA       ---------------------------------------------------
HliNirK      ---------------------------------------------------
NbNirK       ---------------------------------------------------
NaNirK       ---------------------------------------------------
HhNirK       ---------------------------------------------------
HasNirK      ---------------------------------------------------
HaeNirK      ---------------------------------------------------
SsNirK       ---------------------------------------------------
HlNirK       ---------------------------------------------------
HpNirK       ---------------------------------------------------
HaNirK       ---------------------------------------------------
HmaNirK      ---------------------------------------------------
HuNirK       ---------------------------------------------------
```

Figure S2. Complete alignment of NirK sequences of *Ralstonia pickettii* (*Rp*NirK; Protein NCBI ID: WP_039373687.1; UniProt: B2UHR8), *Neisseria gonorrhoeae* (*Ng*AniA; Protein NCBI ID: WP_003705926.1; UniProt: Q5F7A4), *Halorubrum lipolyticum* (*Hli*NirK; Protein NCBI ID: WP_049911241.1), *Natonolimnonius barhuensis* (*Nb*NirK; Protein NCBI ID: WP_054863730.1), *Natronococcus amylolyticus* (*Na*NirK; Protein NCBI ID: WP_005555322.1), *Halobiforma haloterrestris* (*Hh*NirK; Protein NCBI ID: WP_089787040.1), *Halopiger aswanensis* (*Has*NirK; Protein NCBI ID: WP_120243376.1), *Haloplanus aerogenes* (*Hae*NirK; Protein NCBI ID: WP_121921915.1), *Salinigranum salinum* (*Ss*NirK; Protein NCBI ID: WP_152041973.1), *Halobellus limi* (*Hl*NirK; Protein NCBI ID: WP_103992402.1), *Haloferax mediterranei* (*Hm*NirK; Protein NCBI ID: WP_004059594.1), *Halogeometricum pallidum* (*Hp*NirK; Protein NCBI ID: WP_049916781.1), *Halogranum amylolyticum* (*Ha*NirK; Protein NCBI ID: WP_170864849.1), *Haloarcula marismortui* (*Hma*NirK; Protein NCBI ID: WP_011224471.1), *Halorhabdus utahensis* (*Hu*NirK; Protein NCBI ID: WP_012795101.1). The TAT consensus sequence [ST]RRxFLK is underlined, twin arginine 'RR' is highlighted in yellow and lipoprotein consensus sequence [LVI][ASTVI][GAS]C is in bold. The catalytic amino acids are highlighted in magenta, whereas amino acids involved in type 1 copper and type 2 copper coordination centers are highlighted in green and cyan, respectively. The consensus sequence SSFHV/I/P is underlined. The percentages of identity of *Ralstonia pickettii* NirK and *Neisseria gonorrhoeae* AniA with *Haloferax mediterranei* NirK are 27% and 36%, with a coverage of 95% and 97%, respectively; while haloarchaeal NirKs presents percentages of identity among them ranged between 49% and 79%, with a coverage between 98% and 100%.

```
HmNirK      1   ------MLSTTRRRTLQLLGLGGVASLAGCASEAPTAAQSLD--------Q--TEEPTPAQQESPKIVEQVAANPTDIP-
NgAniA      1   -----------MKRQALAAMIASLFALAACGGEPAAQAPAETPAASAEAASSAAQATAETPAGELPVIDAVTTHAPEVP-
RpNirK      1   -MSRLHTVRSLMRAGLISVVLGALLA-----GTAARAAS--------------AKLPGDFGPPRGEPIHAVLTSPPLVP-
PtNirK      1   --MKIQPNKHTLWLPLIAI----LFS-----GNLL-------------------------AASNLKTEQAILTPPPMVP-
AxNirK      1   ----MNALRPTL--------LAAALA------FTMAAGTAW-----------------AQDADKLPHTKVTLVAPPQVHP
AcNirK      1   MTEQLQMTRRTMLAGAA---LAGAVA------PLLHTAQAHAAGAA------AAAGAAPVDISTLPRVKVDLVKPPFVHA
AfNirK      1   MAEQMQISRRTILAGAA---LAGALA------PVLATTSAWGQGAV------R--KATAAEIAALPRQKVELVDPPFVHA

HmNirK     64   -DPITRSEPTEVDVTLRPEEVTAEVE-EGVTFTYMTYNGQVPGPFIRVRQGDTVNLTFENPEENSMPHNVDFHAVAGPGG
NgAniA     69   -PAIDRDYPAKVRVKMETVEKTMKMD-DGVEYRYWTFDGDVPGRMIRVREGDTVEVEFSNNPSSTVPHNVDFHAATGQGG
RpNirK     60   -PPVHRNYPAKVIVELEVVEKEMQIS-EGVSYTFWTFGGTVPGSFIRVRQGDTVEFHLKNHPSSKMPHNIDLHGVTGPGG
PtNirK     44   -PAINRDHSAKVVINLETREQVGRIA-DGVEYVFWSFGETVPGSFIRVREGDEIEFNLSNHPSSKMPHNIDLHAVTGPGG
AxNirK     46   HEQATKSGPKVVEFTMTIEEKKMVIDDKGTTLQAMTFNGSMPGPTLVVHEGDYVQLTLVNPATNAMPHNVDFHGATGALG
AcNirK     66   HDQVAKTGPRVVEFTMTIEEKKLVIDREGTEIHAMTFNGSVPGPLMVVHENDYVELRLINPDTNTLLHNIDFHAATGALG
AfNirK     64   HSQVAEGGPKVVEFTMVIEEKKIVIDDAGTEVHAMAFNGTVPGPLMVVHQDDYLELTLINPETNTLMHNIDFHAATGALG

HmNirK    142   GAEATMTNPGETVKIRFKATYPGAYIYHCAVPN-MDMHISAGMFGLILVEPPEGLPE-------VDKEVYIGQHELYTDK
NgAniA    147   GAAATFTAPGRTSTFSFKALQPGLYIYHCAVAP-VGMHIANGMYGLILVEPKEGLPK-------VDKEFYIVQGDFYTKG
RpNirK    138   GAASSFTAPGHESQFTFKALNEGIYVYHCATAP-VGMHIANGMYGLILVEPPEGLPK-------VDHEYYVMQGDFYTAG
PtNirK    122   GAESSFTAPGHTSTFNFKALNPGLYIYHCATAP-VGMHIANGMYGLILVEPKEGLAP-------VDREYYLVQGDFYTKG
AxNirK    126   GAKLTNVNPGEQATLRFKADRSGTFVYHCAPEGMVPWHVVSGMSGTLMVLPRDGLKDPQGKPLHYDRAYTIGEFDLYIPK
AcNirK    146   GGALTQVNPGEETTLRFKATKPGVFVYHCAPEGMVPWHVTSGMNGAIMVLPRDGLKDEKGQPLTYDKIYYVGEQDFYVPK
AfNirK    144   GGGLTEINPGEKTILRFKATKPGVFVYHCAPPGMVPWHVVSGMNGAIMVLPREGLHDGKGKALTYDKIYYVGEQDFYVPR

HmNirK    214   KAGKKGK-------H-NFDFEAMRNEEPTYVVMNGEKYAWTDAGRGPAATVNTGETVRVFFVDGGPNLSSSFHPIGSVWE
NgAniA    219   KKGAQGL-------Q-PFDMDKAVAEQPEYVVFNGHVGAIAGD---NALKAKAGETVRMYVGNGGPNLVSSFHVIGEIFD
RpNirK    210   KYREKGL-------Q-PFDMEKAIDERPSYVLFNGAEGALTGD---KALHAKVGETVRIFVGNGGPNLVSSFHVIGAIFD
PtNirK    194   EFGEAGL-------Q-PFDMAKAIDEDADYVVFNGSVGSTTDE---NSLTAKVGETVRLYIGNGGPNLVSSFHVIGEIFD
AxNirK    206   GPDGKYKDYATLAESYGDTVQVMRTLTPSHIVFNGKVGALTGA---NALTAKVGETVLLI--HSQANRDTRPHLIGGHGD
AcNirK    226   DEAGNYKKYETPGEAYEDAVKAMRTLTPTHIVFNGAVGALTGD---HALTAAVGERVLVV--HSQANRDTRPHLIGGHGD
AfNirK    224   DENGKYKKYEAPGDAYEDTVKVMRTLTPTHVVFNGAVGALTGD---KAMTAAVGEKVLIV--HSQANRDTRPHLIGGHGD

HmNirK    286   TLYPDGSLSTDPQTHIQTRLVPPGSTTVATMSSPVPGDFKLVDHSLSRVTRKGCMAVIRAEGPEDPEIFDPNPE------
NgAniA    288   KVYVEGGKL--INENVQSTIVPAGGSAIVEFKVDIPGNYTLVDHSIFRAFNKGALGQLKVEGAENPEIMTQKLSDTAYAG
RpNirK    279   QVRYEGGTN--VQKNVQTTLIPAGGAAVVKFTARVPGSYVLVDHSIFRAFNKGALAILKIDGPESKLVYSGKELDSVYLG
PtNirK    263   TVYVEGGSL--KNHNVQTTLIPAGGAAIVEFKVEVPGTFILVDHSIFRAFNKGALAMLKVEGPDDHSIFTGKTAENVYLP
AxNirK    281   WVWETGKFANPPQRDLETWFIRGGSAGAALYTFKQPGVYAYLNHNLIEAFELGAAGHIKVEGKWNDDLMKQIKAPAPIPR
AcNirK    301   YVWATGKFRNPPDLDQETWLIPGGTAGAAFYTFRQPGVYAYVNHNLIEAFELGAAGHFKVTGEWNDDLMTSVVKPASM--
AfNirK    299   YVWATGKFNTPPDVDQETWFIPGGAAGAAFYTFQQPGIYAYVNHNLIEAFELGAAAHFKVTGEWNDDLMTSVLAPSGT--

HmNirK          --------------------------------------------------------------------------------
NgAniA    366   SGAASAPAASAPAASAPAASAS--------EKSVY---------------------------------------------
RpNirK    357   DRAAPNMSAVTKATEASVSGTLTVQDQVQAGRALFAGTCSVCHQGNGAGLPGVFPPLAKSDFLAADPKRAMNIVLHGLNG
PtNirK    341   EGSAIQSLDNTF----TKITANNKDEQIRFGQRVYEANCMACHQANGEGIPGAFPPLAKSDYLNNNPLLGVNAIIKGLSG
AxNirK    361   --------------------------------------------------------------------------------
AcNirK          --------------------------------------------------------------------------------
AfNirK          --------------------------------------------------------------------------------

HmNirK                           ---------------------------------------------------------------
NgAniA                           ---------------------------------------------------------------
RpNirK                437        KIKVNGQEYDSVMPPMTQLNDDEVANILTYVLNSWDNPGGRVSAEDVKKVRAQPAPAKAVAEH
PtNirK                417        PIKVNNVNYNGVMPAM-NLNDEDIANVITFVLNNWDNAGGKVSAEQVAKQRK-----------
AxNirK                           ---------------------------------------------------------------
AcNirK                           ---------------------------------------------------------------
AfNirK                           ---------------------------------------------------------------
```

Figure S3. Complete alignment of NirK sequences of *Alcaligenes xilosoxidans* (*Ax*NirK; Uniprot: O68601), *Achromobacter cycloclastes* (*Ac*NirK; Uniprot: P25006), *Alcaligenes faecalis* (*Af*NirK; Uniprot: P38501), *Haloferax mediterranei* (*Hm*NirK; Protein NCBI ID: WP_004059594.1), *Ralstonia pickettii* (*Rp*NirK; UniProt: B2UHR8), *Pseudoalteromonas translucida* (*Ph*NirK; Uniprot: Q3IGF7), *Neisseria gonorrhoeae* (*Ng*AniA; UniProt: Q5F7A4). The linker loop is highlighted in yellow whereas coordination patterns around catalytic His residue, TRPHL and SSFHV/I/P, are in underlined.

```
HmNosZ     1 MSHNTSTP----DDESNESQQDPLSEYEAQLDAVLEKVDETAGE----ETDVSLELPTLGLSRRDFMKAGAAVGAM---G
WsNosZ     1 --------------------------------------------------------------MQRLLKQSLVVTAS---L
MhNosZ     1 ----------------------------------MKKR--------DDLTKDTPEVSEGGLSRRRFMGAAALAGVA----
HaiNosZ    1 MTQHTQR-PTS--------------------------DEEPDEQ--QSGAADGFDSLLPGLRRRDFMKAGAAVGGL---S
NaNosZ     1 MTKSHASEP----------------------------TTPEEPT--STTAVDDRDPLFARIPRRDFMIAGAATGMM---G
NhNosZ     1 MTDTHAS------------------------------DTNGRST--ETTAVDDRDPLFARIPRRDFMKAGAAAGAM---S
NbNosZ     1 MTDTHD-------------------------------TAGGNST--RTAVEDDRDPLFARIPRRDFMKAGAAAGAI---G
HhNosZ     1 MSDTTPT------------------------------DDRDAID--SETAAEKRDPLFSRIPRRDFMKAGAAAGAM---G
NeNosZ     1 ----------M-----------------------TETNSPT-ES--TLDTDSERDPLFARIPRRDFMKAGAATGAM---A
NaiNosZ    1 MTQTHDSDDSS-----------------------LETNRATDST--TDDDSSERDPLFARIPRRDFMKAGAATGAM---A
HaeNosZ    1 ---MTDTEPDAGEMPEPDPAEKLLREHEAQLDELIADVEPPEDD--ATDDGVTLDLPGLTLGRRDLMKTGAAVGVA---T
HbNosZ     1 MSSHDTSEGEGTDTKTERSAETLLSEHEAQLNALIADVQGPDAD----AVETQLPNLGLGLGRRDFMKAGAAVGVL---G
HpeNosZ    1 MSTNTD--TDEEERTERKDSEELFAEHEAQLNELLADVEGPDAP----EAESTLPSLGDGVTRRDVMKAGAAVGAV---G
HmaNosZ    1 ----------MSKHETPRDPNEVLEEYEETLDSVLAEVESPDEA--TTDDDISLGLPGLSLGRRDFMKAGVAAGAM---G
HgNosZ     1 -------MSDSTSSADSTDPERVVEEYENQLEDVLADVEET---TPRTDDDLSLELAGLQLDRRDFLKAGAVAGAM---T
HcNosZ     1 ---------MSNDANDSSDAESLVEEHEQRINDLVADVDDPAELGADEDDGFSMQLAGLELDRRDFMKAGAATGLASAAG

HmNosZ    70 SIAGCTSLAGNDG-----AGGTTTPHSNSGDVPDHFVPPGEHDEYYGFWSGGHSGDIRIYGLPSMRELTRIPVFNPEQAK
WsNosZ    16 LALGTASLASSDLQTI--MKERKLTEKDVLAAAKTYQPSGRKDEFVVFSSGGQSGQILVYGVPSMRIYKYIGVFTPEPWQ
MhNosZ    35 ---GATGLGTSVMSR------ETWAAAAEEARNKAHVAPGELDEYYGFWSGGHQGEVRVLGVPSMRELMRIPVFNVDSAT
HaiNosZ   49 GLAGCTSLLSEEEEG-----------TVS-AAPDHSIPPGEMDEYYAFLGGGQSGDIRVVGLPSMRELIRIPVFQRDSGR
NaNosZ    48 SLAGCTGLLSDDEQ-------------VSAADVDTEVPPGEHDDYYAFLSGGHSGDIRVYGVPSMRQLMRIPVFNVESAR
NhNosZ    46 SLAGCTGLLDSDDM-------------PAAADVDASVPPGELDDYYAFLSGGHSGDIRVYGVPSMRQIMRIPVFNVESAR
NbNosZ    45 SLAGCTNLLDEGET-------------TAAADVDASVPPGELDEYYAFLSGGHSGDIRVYGVPSMRQIMRIPVFNVESAR
HhNosZ    46 SLAGCTGLLSDDDDD-----------LAAAGDVDSYVPPGEHDDYYAFLSGGHSGEIRVYGIPSMRQLMRIPVFGRESAR
NeNosZ    42 SLAGCTGLLGGDES-------------TSLEDVKTDVKPGDHDDYYAFLSGGHTGEIRVYGLPSMRQLMRIPVFGRESAR
NaiNosZ   53 SMAGCTGLLGGDDS-------------VSLEDVKTSVEPGEHDDYYAFLSGGHTGEIRVYGLPSMRQLMRIPVFQTESAR
HaeNosZ   73 SLAGCSFGGTGGGN----------GNGNGASTTDHKVPPGEHDDYYGFWSGGHSGEVRVVGIPSMRELRRIPVFQKDGAV
HbNosZ    74 SVAGCAGQGDS-------QGGTATPSSSHGDVPDHFVPPGEHDKYYGFWSGGHSGEVRVMGIPSMRELTRIPVFNRECAK
HpeNosZ   72 GLAGCAGLGNN----------TATESNSGGDVPDHKVPPGEHDEYYGFWSGGHAGDIRVYGIPSMRQLTRIPVFNRESAK
HmaNosZ   66 SVAGCSALS--GGD---SSAG--SQSTPSSSGASHTVEPGEHDEYYGFWSGGHSGELRVIGIPSMRELTRIPVFNTDCAS
HgNosZ    68 GLAGCAGSLPTGGDG------SNAANTGSDDVPEHRVPPGEHDEYYGFWSGGHSGEIRVLGIPSMRELTRIPVFNTEGAR
HcNosZ    72 AFAGCQTALPDGKTPSDSGGGGDGGSQGASASHGHFVPPGEKDEYYGFWSGGHSGEIRVIGIPSMRELQRIPVFNTEPAR

HmNosZ   145 GYGFDNQTTEMLESAGD------YTWGDSHHPSLSETDGKYDGKYLFVNDKAHGRVARVNLKYFETDAITNIPNVQSVHG
WsNosZ    94 GYGFDDDSKKVLRQGDIRG--REINWGDTHHPNFTEKNGEYVGDYLFINDKANPRIAVVNLHDFETTQIVVNPIMKSEHG
MhNosZ   106 GWGITNESKEILGGDQQ------YLNGDCHHPHISMTDGRYDGKYLFINDKANTRVARIRLDIMKTDKITHIPNVQAIHG
HaiNosZ  117 GYGHDDESREMLEEAGG------YTWGDTHHPRISQTDGDYDGRFAYVNDKANGRMARIDLTYFETDAIVDIPNQQGTHG
NaNosZ   115 GYGFDDETHEMLQESGG------YTWGDTHHPRVSQTDNEYDGEWLFVNDKANGRMARIDLKYFETDAIIDLPNHQGTHG
NhNosZ   113 GYGFDDETHDMLQDAGG------YTWGDTHHPRVSQTDNEYDGEWLFVNDKANGRMARVDLEYFETDAIIDLPNQQGTHG
NbNosZ   112 GYGFDDETHEMLQEAGG------YTWGDTHHPRVSQTDNQYDGEWLFVNDKANGRMARIDLEYFETDAIIDLPNQQGTHG
HhNosZ   115 GYGYDDRTSEMLEEAGG------YSWGDTHHPRVSQTDNDYDGRWAFVNDKANGRMARIDLEYFETDAIVDIPNQQGTHG
NeNosZ   109 GYGYDDRTSEMLEDAGG------YSWGDTHHPRVSQTDNDYDGRWAFVNDKANGRMARINLKYFETDAIVDIPNQQGTHG
NaiNosZ  120 GYGYDDRTSEMLEEAGG------YTWGDAHHPRVSQTDNDYDGRWAFVNDKANGRMARIDLEYFETDAIVDIPNQQGTHG
HaeNosZ  143 GYGHDEQTKEVLREGGDVGSVAGHEWGDTHHPILSESGGDYDGRYLWINDKVSGRLARINLKYFETDAITDIPNMQACHG
HbNosZ   147 GYGFDDGTGEMLKDAGD------YTWGDSHHPSLSETDGKYDGEYLFVNDKANGRVARVNLKYFETDAILDVPNVQSVHG
HpeNosZ  142 GYGHDDRTTEMLEEAGD------YTWGDSHHPSLSETDGKYDGEYIFVNDKANGRVARVNLTYFETDAIVDIPNVQSVHG
HmaNosZ  139 GYGFTDGTQEMLEEGGG------YSWGDNHHPNLSETDGDYDGEYLYVNDKANGRIARVNLTYFETDAITDVPNMQAIHG
HgNosZ   142 GYGYDDQTDEMLEEAGD------YTWGDNHHPNLSETDGKYDGEYLYVNDKANGRIARVNLKYFETDAITDVPNVQAVHG
HcNosZ   152 GYGFDDQSSEMLEQAGD------YTWGDNHHPNLSETDGDYDGEYLYVNDKANGRIARVNLKYFETDAIVDVPNVQCIHG

HmNosZ   219 CCIQ-SPDTEYVFANSEFRTPLPNDGR-DINNPDKYVSLFTALD--------------PDSMEVLWQVEVDGNLDILDTD
WsNosZ   172 GSFV-TPNTEYVIEASQYAAPLDHQYH----PIEEYEAVFRGAVTLWKFDYAKGKIDEKASFSLEFPPYM-QDLSDAGKG
MhNosZ   180 LRLQKVPKTNYVFCNAEFVIPQPNDGT-DFSLDNS-YTMFTAID--------------AETMDVAWQVIVDGNLDNTDAD
HaiNosZ  191 SCAQ-LPDTDLIFGVGEFRAPIPNDGTGDLHDPDEYGAVLAAID--------------PESMNVEWEVLVDGNMDNGDGS
NaNosZ   189 ACML-MPDSRYVFGVGELRVPIPNDGR-DLDDPSEYGSTLSAMS--------------PDPFDHEWDVRVDCNLDNGDSG
NhNosZ   187 ACAL-MPDTRLIFGVGELRVPIPNDGR-DLEDPSEYGSTLSAMS--------------ADPFDHEWDVRVDCNLDNGDSG
NbNosZ   186 ACAL-MPDTRLIFGVGELRVPIPNDGR-DLEDPSEYGSTLSAMS--------------ADPFDHEWDVRVDCNLDNGDSG
HhNosZ   189 ACCL-LPDTKYVFGVGEFRVPMPNDGQ-DLDDPENYTSTIAAIN--------------PETMNVEWEVLVDGNMDNGDGS
NeNosZ   183 ACCL-LPDTKYVFGVGEFRVPMPNDGQ-DLTDPENYTSVIAAIN--------------PETMNVEWEVLVDGNMDNGDGG
NaiNosZ  194 ACCL-LPDTKYVFGVGEFRVPMPNDGQ-DLDDPSNYTSTIAAID--------------PETMNVEWEVLVDGNMDNGDGG
HaeNosZ  223 CSVQ-SPDTKYVYGVGEFRVPVPNDGTTDPDDADSYWSVFAALD--------------PESMEVVWQVRVSGNLDNADSD
HbNosZ   221 CCIQ-SPDTEYVFANGEFRAPLPNDGR-DVDDPSKYVSLFSAID--------------PESMDVLWQVKVSGNLDIADSD
HpeNosZ  216 CCIQ-SPDTGYVFANSEFRTPMPNDGR-DVNEPEEYVSVFSALD--------------PESMDVLWQVEVDGQLDIVDSD
HmaNosZ  213 CCVL-SPDTKYVLGNGEFRAPLPNDGT-DAKNPDNYTSLFVAVD--------------PDSMETQWQVKVDGNLDIVDTG
HgNosZ   216 TCVL-SPDTKYILGNGEFRTPLPNDGR-DLDNPENYTSLLSAVD--------------PESMETKWQVKVDGNLDIIDSG
HcNosZ   226 TTVL-SPDTKYVLGNGEFRAPLPNDGR-DVNNPEEYTALFSAVD--------------PESMETAWQVKVDGNLDIVDTG
```

```
HmNosZ   283 KDGRWAISSAYNDEEGV---------EIEEMTKNDRDFVKAFDVPAIQKAVDA-GNYKKVN--GIPVVDGTKESSLN-KG
WsNosZ   246 ESFGWAFTNSFNSEMYTGGIEKGLPPFEAGMSRNDTDYMHVYNWQMLEKLAQDPKNYKIYH--------GHRVISIEAAV
MhNosZ   244 YTGKYATSTCYNSERAV---------DLAGTMRNDRDWVVVFNVERIAAAVKA-GNFKTIGDSKVPVVDGRGES------
HaiNosZ  256 KEGRYFFTTGYNSEGAV---------TEKGMTRSDRDDVKAFDIPRIEAAVEA-GDYETIN--EVPVVDGRKDSSLN-QG
NaNosZ   253 KDGEWFFTTSYNSEEGV---------TEREMTAADTDYVVAFNIPRIEDAIDA-GEYEEIN--GVPVVDGTEDSSLN-DG
NhNosZ   251 KNGEWFFTTSYNSEEGV---------TEAEMTAADTDYVVAFNIPRIEAAVDA-GEYETIN--GVPVVDGTEDSPLN-TG
NbNosZ   250 KYGEWFFTTSYNTEEAV---------TESEMTASDTDYVVAFNIPRIEDAVDA-GEFEEIN--GVPVVDGTEDSPLN-DG
HhNosZ   253 KQGRWFFATGYNSESAT---------TESGMSSSDTDWVKAFDVPAIEDAVEA-GEYEEIN--GVPVVDGTRDSALN-SG
NeNosZ   247 KEGRWFFTTGYNSEHAT---------TQSEMSSSDTDWVKAFDVPAIEEAVEA-GEYDEMN--GVPVVDGTRDSALN-EG
NaiNosZ  258 KEGRWFFATGYNSEHAT---------TESEMSSSDTDWVKAFDVPAIEEAVEA-GDYDEIG--GVPVVDGTRDSSLN-ES
HaeNosZ  288 KEGNWAFATGYNKEEAF---------EIDGMTHDDRDNLKAFDIEAIEAALDA-GEAEEIN--GVPVLDGRQDSPLT-SG
HbNosZ   285 KDGRWLLSSSYNDEEGV---------EIEEMTRDDRDSVKAFDVPAIQKAVDA-GKYEEVN--GVPVVDGTRESSLN-KG
HpeNosZ  280 KDGRWVLSSSYNDEEGV---------EVEEMTKDDRDDVKAFDVPAIQELVDA-GEYEEVN--GVPVVDGTKESPHN-EG
HmaNosZ  277 KEGRWAISSAYNSEEAT---------DIQGMTKDDRDNVKAFDIPAIEQAVEN-GNYEEVN--GIPVVDGTQGSSLN-QG
HgNosZ   280 KEGRWAISSAYNSEEAV---------EIEGMSHDDRDYVKAFDIPAIEQAVER-GKYEEVN--GVPIVDGTQSSTLN-KG
HcNosZ   290 KEGRWAISSCYNSEEAT---------EIQGMTRDDRDYAKVFDIPAIEQAVEN-GNYEEVN--GVPVVDGTRESDLN-SG

HmNosZ   350 DNPIVRYVPTPKSPHCVEVEPNGKYAMVAGKLSPTVSIIDIEKLGTAS----------------DPKDTIVGQPKLGLGP
WsNosZ   318 KAGALFLIPEPKSPHGVDVSPDGRYIVVGGKLDTHASVYDFRKIKQLIDKKEFIGADPYGIPILDMKKTLHGQVELGLGP
MhNosZ   308 --EFTRYIPVPKNPHGLNTSPDGKYFIANGKLSPTVSVIAIDKLDDLFED------------KIELRDTIVAEPELGLGP
HaiNosZ  323 DDPVVHYIPTPKSPHGISVTPDNKYAIASGKLDPTASVIQIDKIDEVD----------------DPTDAIVGQPKLGLGP
NaNosZ   320 DEPLVRYIDVPTNPHGVSVTPDGRYAIASGKLDPTCTVIEIDRLNEVD----------------DPNDAIVGRVNVGNGP
NhNosZ   318 SEPLVRYIDVPTNPHGVSVTPDGQYAIASGKLDPTCTIIEIDQLTEVD----------------DPNDAIVGRVNVGNGP
NbNosZ   317 DEPLVRYIDVPTNPHGVSVTPDGEYAIASGKLDPTCTIIEVDQLNEVD----------------DANDAIAGRVNVGNGP
HhNosZ   320 DRPIVRYVDVAKSPHGVSVTPDNQYAIASGKLDPTATVIDIEQLAEVD----------------DPNDAIVARPTLGMGP
NeNosZ   314 DRPIVRYIDVSKSPHGVSVTPDGQYAIASGKLDPTATVIDIETLAEAD----------------DPNESIVGRPRLGMGP
NaiNosZ  325 DRPIVRYLDVSKSPHGVSVTPDGKYAIASGKLDPTASVIDIETLADAD----------------DPNDAIVGRPRLGMGP
HaeNosZ  355 SDPIVHYIPTPKGPHGCDVEPSGTYVTASGKLSPTVTMVEIDKIKEVD----------------DPEDAIVGQPRVGMGP
HbNosZ   352 DNPLVRYIPTPKSPHCVEVTPDGKYGIVAGKLSPTVSIIDIEKLGTEP----------------DPAKTIVGQPKLGLGP
HpeNosZ  347 DDPIVRYIPTPKSPHCVEVTPDGKYGMVAGKLSPTVSIIDIEKLATAD----------------DPADTIVGQPKVGLGP
HmaNosZ  344 DRPVVKYIPTPKSPHCVEVGPNGDYAFIAGKLSPTVTMLDLNALADSS----------------DPDEVVAGRPRVGLGP
HgNosZ   347 DRPIVRYVPTPKSPHCVEVEPNGDYAMIAGKLSPTVTILDIDKLGEVS----------------DPADAVVGRPKVGLGP
HcNosZ   357 DEPLVRYISTPKSPHCVEVGPDGKYAFIAGKLSPTVTMLDLDKIGEVD----------------DPADAVAGRPKVGLGP

HmNosZ   414 LHTTYDGR-GHAYTSLFIDSQVVKWDIETAINSPK--------KSEDAILGKIDVHYNPGHIQAIQAMSTEPTGDWLVVL
WsNosZ   398 LHHTYDAQDGIIYTSLYVDSQIVKWDYKNL-----------------KVLDRVNVHYNIGHLDSMEGKSAKPKGKYALAL
MhNosZ   374 LHTTFDGR-GNAYTTLFIDSQVCKWNIADAIKHYNG-------DKVNYIRQKLDVQYQPGHNHASLTESRDADGKWLVVL
HaiNosZ  387 LHTAYDGR-GHAYTSLFIDSQVVKWDIEEAVEADA--------GSESPVIEKIDVHYNPGHLIASESYTEDPAGDWLISL
NaNosZ   384 LHTAYDGR-GHAYTTLFVDSQVVKWDIEAAVEAEK--------GSEDPVIEKHDVHYSPGHLIAAESYTGDPQGDWLIVL
NhNosZ   382 LHTAYDGR-GHAYTTLFVDSEVVKWDIDAAVEAEM--------GSADPVIQKHEVHYSPGHLIAAESYTGDPQGDWLIVL
NbNosZ   381 LHTAYDGR-GHGYTTLFVDSQVVKWDIEAAVDAEM--------GSADPVIEKHDVHYSPGHLIAAESYTGDPQGDWLIVL
HhNosZ   384 LHTAYDGR-GHAYTTLFIDSQVVKWDIEAAVDAYPDDEPEGGDQESPAVVEKIDVHYNPGHLIAAESYTADPQGDWLISL
NeNosZ   378 LHTAYDGR-GHAYTTLFIDSQVVKWDIEAAVEASE--------ESTDPVIEKIDVHYNPGHLIASESYTADPQGDWLISL
NaiNosZ  389 LHTAYDGR-GHAYTSLFIDSQVVKWDIEAAVEASE--------ESEDPVIEKVDVHYNPGHLIAAESYTADPQGDWLISL
HaeNosZ  419 LHTTWDGR-GHGYTTLFIDSQIAKWDIEQAVEAEK--------GSEEPVVGTIDVHYNPGHLQAVEAETTDPAGDWLVTL
HbNosZ   416 LHTTYDDR-GHGYTSLFIDSQVVKWDIEKAVESPK--------GSEEAILGKIDVHYNPGHIQAVQAMSVEPTGDWLVVL
HpeNosZ  411 LHTTYDDR-GHAYTSLFIDSQVVKWDIEEAVNAEK--------GSEEPILGKIDVHYNPGHIQAVQAMSTEPEGNWLVAL
HmaNosZ  408 LHTTFDGN-GHAYTSLFIDSQTVKWDIQAAVEAEE--------GSEDSIIEKQDVHYNPGHIQALEAMTTDPDGEWLVSL
HgNosZ   411 LHTTFDGN-GHAYTSLFIDSQAVKWDIEKAVEAEE--------GSTDPVIEKIDVHYNPGHIQAVKAMTTDPDGEWLISL
HcNosZ   421 LHTTFDGN-GHAYTSLFIDSQVAKWDIEEAANAEL--------GSSDPVIEKQDVHYNPGHIQALEAMTTDPDGEWLISL

HmNosZ   485 NKLSKDRFLPVGPIYPDNDQLIYIGNDKDDETGGMELVSDHPV-YPEPHDAIFAAKDKIQPAKTWDAADYEG-------E
WsNosZ   461 DKLSIDRFNPVGPLHPQNHQLIDIGGP------KMELIYDLPIPLGEPHDVISIAADKLKPQVTYPMGTNSR----TGKQ
MhNosZ   446 SKFSKDRFLPVGPLHPENDQLIDISGE------EMKLVHDGPT-YAEPHDCILVRRDQIKTKKIYERNDPYFASCRAQAE
HaiNosZ  458 NKLSKDRFLPVGPQHPENDQLIYIGDDE----AGMEHVKDSPA-HAEPHDASICHRSKLDPAKTYDPDDLDL--------
NaNosZ   455 NKLSKDRFLPVGPVFPENDQLFYIGDDD----AGMELVKDTPT-YPEPHDASIVRADRLDPASVYDPDDLEL--------
NhNosZ   453 NKLSKDRFLPVGPVFPENDQLFYIGDDD----AGMELVKDTPA-YPEPHDASIVRADRLDPASVYDPDDLTL--------
NbNosZ   452 NKLSKDRFLPVGPVFPENDQLFYIGDDD----AGMELVKDTPA-YPEPHDASIVRADRLDPASVYDPDDLAL--------
HhNosZ   463 NKLSKDRFLPVGPMHPENDQLIYIGDDE----EGMKLVKDTPS-YAEPHDASIVSADKLDPAKVYDPEDYDE--------
NeNosZ   449 NKLSKERFLPVGPMHPENDQLIYIGDDE----EGMSLVKDTPS-YAEPHDASIVSAEKLDPAKVYDPEDYDE--------
NaiNosZ  460 NKLSKDRFLPVGPMHPENDQLIYIGDDE----EGMSLVKDTPS-YAEPHDASIVSAEKLDPAKVYDPEDYDE--------
HaeNosZ  490 NKLSKDRFISVGPIHPDNDQLIAIGEDAETETGGMELVADHPV-HPEPHDCVFASRDKISPNNIWDREDYEG-------E
HbNosZ   487 NKLSKDRFLPVGPIHPDNDQLIYIGNDKDNETGGMELVSDHPV-YPEPHDAIMVHKDKLDPAKIWDAKDYEG-------E
HpeNosZ  482 NKLSKDRFLPVGPIYPDNDQLIYIGDKDDETGGMELVADHPV-YPEPHDAIFAHKDKIEPATTWDPEDYDE--------
HmaNosZ  479 NKLSKDRFLPVGPIMPDNDQLIHIGQGE----KEMELVADHPA-YPEPHDCVFAHKDKIDAKKVYDKDDYE---------
HgNosZ   482 NKLSKDRFLPVGPIHPDNDQLIHIGQGE----KEMELVADHPA-YPEPHDCVFADAETISPATTWDKEDYAG-------E
HcNosZ   492 NKLSKDRFLPVGPIHPDNDQLIHIGDGE----KEMEIVADHPA-YPEPHDCVFAHRDKINPATTWDKADYEG-------E
```

```
HmNosZ   557 KEYVKESNS-RVERIDEETVEVYTSVKRSEYGLRDFTVKEGDEVTLTATNIEGSQDIVHGLAIPEHNVHLALAPQDTREA
WsNosZ   531 HEAMTLAGQE-RVERKGNEVKIYGTLIRSHINPEHVTVNKGDKVTFYLTNLERAQDETHGFAVSGYNVHASVEPGKTVAV
MhNosZ   519 KDGVTLE-SDNKVIRDGNKVRVYMTSVAPQYGMTDFKVKEGDEVTVYITNLDMVGDVTHGFCMVNHGVSMEISPQQTASV
HaiNosZ  525 -EYTDEGEE-TMERVGDDRVEIEMYSTRNKYGFQEMTVTEGDTIEMQVTNIETTSDMLHSVAIPEHDVHMRVAPQETRKV
NaNosZ   522 -DFISADDEENFIERDGDQVRVEMYNQRNHFGFEDITVREGDEVTIRSTNIESEEDIIHSLAIPQHDVNVKLAPQETREV
NhNosZ   520 -DFISTDDEDNFIERDGDRVHVEMHSQRNYFSFEDIAVQEGDAVTIRTTNIEQTEDMLHSVAIPEYDINVKIAPQETREV
NbNosZ   519 -EFISPDDEDNFIERDGDRVHVEMYNARNTFGLEDIVVQEGDEVTIRSTNIEQEEDILHSVAIPQHNVNIKLAPQETREV
HhNosZ   530 -EYIEPDASD--IIREDGHVHVKMHSQRNEFGFQEVTVQEGDEVTFTVTNIEQTPDVLHSLAIPEHDVNIKLAPQETREV
NeNosZ   516 -EFVDTEDVD--ISREDGRVHVKMYSTRNEFGFEEVTVTEGDEVTMTVTNIEQTPDILHSIAIPEHDINMKLAPQETREV
NaiNosZ  527 -EFISPEDND--ISRENGRVHVKMHSQRNQFGFTDVTVTEGDEVTFTVTNVEQTADLLHSLAIPEYDVNIKLAPQETREV
HaeNosZ  562 KEYVTEDNS-RVERTGDRSVEVYTSVKRSEYGLRDFTVKEGDEVTITVTNIESSRDIIHGLAIPQYAINLSIAPQDTRKV
HbNosZ   559 REYISEKNS-GIERLDDSTVEVNMSSKRSEFGLREFTVKEGDEVQMTVTNIEKSPDIIHGVAIPEHDINLALAPQDTREV
HpeNosZ  553 -EFVSEEDS-RVERVDDSTVEVYASVKRSEYGLRDFTVQEGDEVTLTATNIEGTQDIVHGLAIPEHDVNLALAPQDTRQV
HmaNosZ  545 ETYITEEDS-GVERTGENSVHVKMTTKRSEFGLPDFTVQEGDEVKLSTTNIEGVQDIIHGVAIPEHDINYAVAPQDTREV
HgNosZ   550 KPFVTEKDS-GVERTGEQSVHVKSSVKRSEYGMSEFTVKEGDEVTLTVTNIEDVRDVIHGVAIPEHDVNLAIAPQDTREV
HcNosZ   560 KPFVTAENS-GVERTGENSVHVKASSKRSEYGMYDFTVQEGDEVRLTVTNVEGVRDIIHGVAIPEHDVNLAVAPQDTREV

HmNosZ   636 TFTADKPGVYWIYCTYFCSALHLEMRSRMIVEPRT---------------------------------------------
WsNosZ   610 TFTADEEGVFPYYCTEFCSALHLEMMGYLYVKDPKKKYESVKELKLQKMSKEQLESEYKKVIATNKATDDVIQSVVKFLK
MhNosZ   598 TFTAGKPGVYWYYCNWFCHALHMEMGGRMLVEKA----------------------------------------------
HaiNosZ  603 TFTADDPGVYWIYCAHFCSALHLEMRSRLIVEPEE---------------------------------------------
NaNosZ   601 TFTADEPGVYWIYCAYFCSALHLEMRSRLLVEPAD---------------------------------------------
NhNosZ   599 TFTADKPGVYWIYCAFFCSALHLEMRSRLLVEPAD---------------------------------------------
NbNosZ   598 TFTADEPGVYWIYCAYFCSALHLEMRSRLLVEPAD---------------------------------------------
HhNosZ   607 TFTADEPGVYWMYCAFFCSALHLEMRSRLIVEPAE---------------------------------------------
NeNosZ   593 TFTADEPGVYWMYCAFFCSALHLEMRSRLIVEPAE---------------------------------------------
NaiNosZ  604 TFTADEPGVYWMYCAFFCSALHLEMRSRLLVEPAE---------------------------------------------
HaeNosZ  641 TFTADEPGIYWAYCTYFCSALHLEMRSRMIVEPRD---------------------------------------------
HbNosZ   638 TFTADDPGVYWIYCTYFCSALHLEMRSRMIVEPRD---------------------------------------------
HpeNosZ  631 TFTADEPGVYWIYCTYFCSALHLEMRSRMIVEPSE---------------------------------------------
HmaNosZ  624 TFTADDPGVYWIYCTYFCSALHLEMRSRMIVEPAEG--------------------------------------------
HgNosZ   629 TFTADEPGVYWIYCTYFCSALHLEMRSRMLVEPRE---------------------------------------------
HcNosZ   639 TFTADDPGVYWIYCTYFCSALHLEMRSRMIVEPSE---------------------------------------------

HmNosZ       --------------------------------------------------------------------------------
WsNosZ   690 DKNYAKYPKVKSLVEDALDQYGKIGEVKAKADESYKKGDVNGAILWEYQVWQYMVKTADVGLRAKNNLAKELATPMKPAA
MhNosZ       --------------------------------------------------------------------------------
HaiNosZ      --------------------------------------------------------------------------------
NaNosZ       --------------------------------------------------------------------------------
NhNosZ       --------------------------------------------------------------------------------
NbNosZ       --------------------------------------------------------------------------------
HhNosZ       --------------------------------------------------------------------------------
NeNosZ       --------------------------------------------------------------------------------
NaiNosZ      --------------------------------------------------------------------------------
HaeNosZ      --------------------------------------------------------------------------------
HbNosZ       --------------------------------------------------------------------------------
HpeNosZ      --------------------------------------------------------------------------------
HmaNosZ      --------------------------------------------------------------------------------
HgNosZ       --------------------------------------------------------------------------------
HcNosZ       --------------------------------------------------------------------------------

HmNosZ       --------------------------------------------------------------------------------
WsNosZ   770 QKGEEAYLKGGCNGCHVIGQVSSGPDLTGVLSRHENAEKWVFDFIKNPASKYEEDYVKTMINYFNLRMPNQHMNDQEIKD
MhNosZ       --------------------------------------------------------------------------------
HaiNosZ      --------------------------------------------------------------------------------
NaNosZ       --------------------------------------------------------------------------------
NhNosZ       --------------------------------------------------------------------------------
NbNosZ       --------------------------------------------------------------------------------
HhNosZ       --------------------------------------------------------------------------------
NeNosZ       --------------------------------------------------------------------------------
NaiNosZ      --------------------------------------------------------------------------------
HaeNosZ      --------------------------------------------------------------------------------
HbNosZ       --------------------------------------------------------------------------------
HpeNosZ      --------------------------------------------------------------------------------
HmaNosZ      --------------------------------------------------------------------------------
HgNosZ       --------------------------------------------------------------------------------
HcNosZ       --------------------------------------------------------------------------------
```

```
HmNosZ          ---------------
WsNosZ     850  IIEYLKWIDENAGLF
MhNosZ          ---------------
HaiNosZ         ---------------
NaNosZ          ---------------
NhNosZ          ---------------
NbNosZ          ---------------
HhNosZ          ---------------
NeNosZ          ---------------
NaiNosZ         ---------------
HaeNosZ         ---------------
HbNosZ          ---------------
HpeNosZ         ---------------
HmaNosZ         ---------------
HgNosZ          ---------------
HcNosZ          ---------------
```

Figure S4. Complete alignment of NosZ sequences of *Wolinella succinogenes* (*Ws*NosZ; Protein NCBI ID: WP_129545366.1; UniProt: Q5F7A4), *Marinobacter hydrocarbonoclasticus* (*Mh*NosZ; Protein NCBI ID: WP_039373687.1; UniProt: B2UHR8), *Halorubrum aidingense* (*Hai*NirK; Protein NCBI ID: WP_007998714.1), *Natronococcus amylolyticus* (*Na*NirK; Protein NCBI ID: WP_049891949.1), *Natrialba hulunbeirensis* (*Nh*NosZ; Protein NCBI ID: ELY91737.1), *Natronolimnobius baerhuensis* (*Nb*NosZ; Protein NCBI ID: WP_087715262.1), *Halobiforma haloterrestris* (*Hh*NosZ; Protein NCBI ID: WP_089784324.1), *Natrinema ejinorense* (*Ne*NosZ; Protein NCBI ID: WP_097381668.1), *Natronorubrum aibiense* (*Nai*NosZ; Protein NCBI ID: WP_152944304.1), *Haloplanus aerogenes* (*Hae*NosZ; Protein NCBI ID: WP_121921888.1), *Haloferax mediterranei* (*Hm*NosZ; Protein NCBI ID: WP_004056356.1), *Halogeometricum borinquense* (*Hb*NosZ; Protein NCBI ID: WP_163487366.1), *Halolamina pelagica* (*Hpe*NosZ; Protein NCBI ID: SFP13007.1), *Haloarcula marismortui* (*Hma*NosZ; Protein NCBI ID: WP_011222995.1), *Hagranum gelatinilyticum* (*Hg*NosZ; Protein NCBI ID: WP_089699362.1), *Halosimplex carlsbadense* (*Hc*NosZ; Protein NCBI ID: WP_006884108.1). The TAT consensus sequence [ST]RRxFLK is underlined and lipoprotein consensus sequence [LVI][ASTVI][GAS]C is in bold. Twin arginine 'RR' and hypothetical amino terminal residue of *Haloferax mediterranei* NosZ are highlighted in yellow and green, respectively. Conserved residues coordinating Cu_A center atoms are highlighted in gray whereas conservative substitutions are highlighted in turquoise. Conserved His residues coordinating Cu_Z center are highlighted in cyan and conserved Lys and Glu are in magenta. The percentages of identity of *Wolinella succinogenes* and *Marinobacter hydrocarbonoclasticus* NosZs with *Haloferax mediterranei* NosZ are 33% and 46%, respectively, with a coverage of 88% and 91% respectively, while haloarchaeal NosZ presents an identity range among them between 50% and 77% (coverage between 93 and 100%).

Table S1. Interaction distance (Angstroms) between the enzyme residues and the metal centres in NarG, NirK and NosZ templates and models.

**PcrA** ***Azospira oryzae***:<br>C64-S-Fe: 2.3<br>C68-S-Fe:2.4<br>H60-N-Fe:2.2<br>C102-S-Fe:2.2	**NarG** ***Haloferax mediterranei***<br>C114-S-Fe: 2.2<br>C118-S-Fe:2.3<br>H110-N-Fe:2.0<br>C153-S-Fe:1.9	
Asp-Mo:2.0<br>Dithiolenes-Mo:2.2, 2.4, 2.4, 2.6	Asp-Mo:2.0<br>Dithiolenes-Mo:2.2, 2.4, 2.4, 2.6	
**NirK** ***Neisseria gonorrhoeae***:<br>H94-N-Cu:2.0<br>H143-N-Cu:1.9<br>C135-S-Cu: 2.1<br>M148-S-Cu:2.6	**NirK** ***Haloferax mediterranei***<br>H129-N-Cu:2.1<br>H179-N-Cu:2.0<br>C170-S-Cu: 2.1<br>M183-S-Cu:2.6	**Type 1 copper site**
H99-N-Cu:2.1<br>H134-N-Cu:2.2<br>H289-N-Cu:2.3	H134-N-Cu:2.3<br>H169-N-Cu:2.3<br>H329-N-Cu:2.0	**Type 2 cooper site**
**NosZ** ***Marinobacter hydrocarbonoclasticus***<br>H576-N-CuA:2.1<br>C611-S-CuA:2.3<br>W613-O-CuA:2.6<br>C615-S-CuA:2.3<br>H619-N-CuA:2.0<br>M622-S-CuA:2.47	**NosZ** ***Haloferax mediterranei***:<br>H614-N-CuA:2.3<br>C649-S-CuA:2.4<br>Y651-O-CuA:2.4<br>C653-S-CuA:2.5<br>H657-N-CuA:1.9<br>M660-S-CuA:2.0	**Cu_A**
H129-N-CuZ:2.2<br>H130-N-CuZ:2.1<br>H178-N-CuZ:2.1<br>H320-N-CuZ:2.3<br>H375-N-CuZ:2.1<br>H426-N-CuZ:2.0<br>H487-N-CuZ:2.0	H168-N-CuZ:2.1<br>H169-N-CuZ:2.3<br>H217-N-CuZ:2.2<br>H364-N-CuZ:2.2<br>H415-N-CuZ:2.1<br>H465-N-CuZ:2.1<br>H532-N-CuZ:1.9	**Cu_Z**
